# The Effectiveness of Transcranial Direct Current Stimulation (tDCS) in Improving Performance in Soccer Players—A Scoping Review

**DOI:** 10.3390/jcm15031281

**Published:** 2026-02-05

**Authors:** James Chmiel, Donata Kurpas

**Affiliations:** 1 Institute of Neurofeedback and tDCS Poland, ul. 3 Maja 25-27, 70-215 Szczecin, Poland; 2 Division of Research Methodology, Department of Nursing, Faculty of Nursing and Midwifery, Wroclaw Medical University, 51-618 Wrocław, Poland

**Keywords:** transcranial direct current stimulation, tDCS, non-invasive brain stimulation, neuromodulation, neurostimulation, football, soccer, sport, neurodoping

## Abstract

**Background/Objectives:** Transcranial direct current stimulation (tDCS) is increasingly used by athletes, yet sport-performance-enhancement findings are mixed and often small, with outcomes depending on stimulation target, timing, and task demands. Aim: This scoping review mapped and synthesized the soccer-specific trial evidence to identify (i) which tDCS targets and application schedules have been tested in soccer players, (ii) which soccer-relevant outcomes show the most consistent immediate (minutes–hours) or training-mediated benefits, and (iii) where evidence gaps persist. **Methods:** We conducted a scoping review of clinical trials in footballers, following review best-practice guidance (PRISMA-informed) and a preregistered protocol. Searches (August 2025) spanned PubMed/MEDLINE, ResearchGate, Google Scholar, and Cochrane, using combinations of “football/soccer” and “tDCS/transcranial direct current stimulation,” with inclusion restricted to trials from 2008–2025. Dual independent screening was applied. Of 47 records identified, 21 studies met the criteria. Across these, the total N was 593 (predominantly male adolescents/young adults; wide range of levels). **Results:** Prefrontal protocols—most commonly left-dominant dorsolateral prefrontal cortex (DLPFC) (+F3/−F4, ~2 mA, ~20 min)—most consistently improved post-match recovery status/well-being (e.g., fatigue, sleep quality, muscle soreness, stress, mood), and when repeated and/or paired with practice, shortened decision times and promoted more efficient visual search. Effects on classic executive tests were inconsistent, and bilateral anodal DLPFC under fatigue increased risk-tolerant choices. Motor-cortex targeting (C3/C4/Cz) rarely changed rapid force–power performance after a single session—e.g., multiple well-controlled trials found no immediate CMJ gains—but when paired with multi-week training (core/lumbar stability, plyometrics, HIIT, sling), it augmented strength, jump height, sprint/agility, aerobic capacity, and task-relevant EMG. Autonomic markers (exercise HR, early HR recovery) showed time-dependent normalization without specific tDCS effects in single-session, randomized designs. In contrast, a season-long applied program that added prefrontal stimulation to standard recovery reported significantly reduced creatine kinase. Across studies, protocols and masking were athlete-friendly and rigorous (~2 mA for ~20 min; robust sham/blinding), with only mild, transient sensations reported and no serious adverse events. **Conclusions:** In soccer players, tDCS shows a qualified pattern of benefits that follows a specificity model: prefrontal stimulation can support post-match recovery status/well-being and decision efficiency, while M1-centered stimulation is most effective when coupled with structured training to bias neuromuscular adaptation. Effects are generally modest and heterogeneous; practitioners should treat tDCS as an adjunct, not a stand-alone enhancer, and align montage × task × timing while monitoring individual responses.

## 1. Introduction

Football match play imposes intermittent, multi-directional loading characterized by frequent transitions between walking, jogging, striding, and brief sprints, layered with accelerations, decelerations, jumps, tackles, and duels [1]. Across elite competition, outfield players typically cover ~10–13 km per match [2], with a small but decisive fraction at high intensity; decisive actions follow short explosive efforts and rapid speed changes rather than steady running [3,4,5]. Positional roles modulate external load—central midfielders accrue the most significant total distance, wide players accumulate more high-speed running, and central defenders perform fewer long sprints but more forceful decelerations and aerial actions [6,7,8,9]. These outputs fluctuate with opponent quality, scoreline, and tactical scheme (e.g., high pressing elevates high-intensity bouts and acceleration counts), underscoring the need for individualized conditioning [10,11,12,13]. Moreover, standard “speed-zone” cut-offs and classic distance metrics under-represent the energetic cost of accelerations/decelerations, which contribute meaningfully to match load [14,15].

Physiologically, football taxes both aerobic and anaerobic pathways. Mean heart rate during matches often sits around ~75–85% HRmax with frequent excursions > 90%, reflecting continuous submaximal activity punctuated by high-intensity bursts [1], and elite VO_2max_ commonly ranges from the mid-50s to low-63 mL·kg^−1^·min^−1^ in male players (somewhat lower, on average, in elite female players) [16,17], supporting rapid recovery between sprints. Blood lactate averages remain modest because of the sport’s intermittent nature, but brief spikes follow intense passages such as pressing sequences [18,19,20]. Muscle glycogen becomes meaningfully depleted—especially in fast-twitch fibers—by the final third, contributing to late-game reductions in high-intensity running and heightened injury susceptibility [21,22]. Environmental stressors interact with these demands: heat generally suppresses total and high-intensity running (despite preserved or slightly higher peak sprint speed). It can alter technical execution, while moderate altitude typically reduces high-intensity running due to lower oxygen availability [23].

tDCS is a non-invasive method that passes a gentle, steady electrical current across the scalp using two or more saline-soaked sponge electrodes—typically one anode and one cathode [24]. It is painless for most people, generally well tolerated, and considered safe, with only a few minor side effects reported [25]. A small battery-powered device delivers direct current, usually 0.5–2 mA, a fraction of which crosses the skull to influence the cortex. The polarity matters: anodal stimulation tends to depolarize neurons and raise their excitability, while cathodal stimulation tends to hyperpolarize them and reduce activity [26,27]. Because current flows between the electrodes, standard tDCS is relatively diffuse, though using smaller electrodes can make the effects more focused [28]. Sessions usually run 15–30 min (20 min is common). The physiological effects outlast the stimulation itself: even ~3 min can produce measurable changes, and 10 or more minutes at 1–2 mA can stabilize those changes for at least an hour [27,29]. A 15 min session can modulate cortical excitability for about 90 min, and repeated sessions extend these effects further [30,31,32]. These after-effects are thought to come from subthreshold shifts in membrane potential and ensuing synaptic plasticity, rather than direct neuronal firing [33]. Indeed, many studies show that tDCS can induce durable, LTP-like or LTD-like changes consistent with Hebbian mechanisms [34]. This plasticity depends heavily on NMDA receptor signaling and factors like BDNF; for example, pairing anodal stimulation with low-frequency input can yield persistent LTP in rodent cortex, an effect abolished by NMDA antagonists [31]. Crucially, context shapes the outcome. The effects of tDCS interact with the brain’s current state and ongoing activity: delivering stimulation at rest versus during a task—or even differences in arousal level or time of day—can weaken or flip the expected excitability changes, a form of metaplasticity [35,36]. And tDCS influences networks, not just a single spot. Perturbing one node (e.g., M1) can propagate through connected circuits [37], and imaging work shows shifts in large-scale functional connectivity, meaning behavior depends on the broader network state [38,39]. In short, the core physiological signatures of tDCS—membrane polarization, changes in excitation/inhibition, and plasticity—are well established, but how they appear in practice depends on the brain’s ongoing dynamics.

tDCS effectively improves numerous parameters of athletic performance, which is crucial for successful competitive soccer performance. Still, the physiological mechanisms of tDCS in enhancing sports performance are quite enigmatic and poorly understood. Several reviews report that tDCS can boost performance in running and cycling [40], endurance and peak force [41], upper-limb motor and endurance tasks [42,43], general exercise output [44], maximal strength and lower-limb explosiveness [45], basketball performance [46], and even both physical and psychological outcomes in national- or international-level athletes [47]. Benefits have also been noted for visuomotor skills [48] and for lowering perceived exertion [49]. That said, the average performance gain appears small and depends on what we measure and how we stimulate. A 2022 meta-analysis of 43 studies found an overall standardized mean difference (SMD) of 0.25 (95% CI 0.14–0.36), with somewhat larger effects for strength (≈0.31) and visuomotor tasks (≈0.29) than for endurance (≈0.18). Results were uneven across studies, meta-regressions did not identify reliable “best” parameters, and moderators such as genetics, sex, and training history likely shape who responds [48]. Other reviews argue that any benefits are modest and may be inflated by low study quality and selective reporting [50]. Overall, the sport-performance literature is mixed and methodologically diverse. While many individual trials report gains, pooled estimates are small, with high between-study variability and signs of publication bias. Most studies are small and underpowered, and responses vary widely—only about 39–45% of participants show the expected excitability or behavioral change in classic M1 protocols. Blinding is also a challenge: a standard sham can have biological effects, and participants often guess their condition above chance. These issues warrant a cautious reading of the claimed benefits [51,52,53].

It is worth noting that tDCS is perceived as a form of “neuro-doping,” a widely available technique that can effortlessly enhance performance. However, it is not currently banned by the WADA. Although tDCS meets two criteria that could lead to a ban (violating the “spirit of sport” and potentially enhancing performance), tDCS is non-invasive, safe, inexpensive, and accessible to everyone. Otherwise, it is no different from other techniques widely used in athletes. For this reason, current recommendations state that the safety of tDCS should be monitored for long-term use in athletes. It is safe to assume that WADA will not ban tDCS anytime soon [54].

Operational definitions used in this review: In the present soccer literature, “recovery” refers to post-match/post-exercise recovery status (i.e., short-term restoration of readiness after match or training load), indexed by multidimensional well-being scales (fatigue, sleep quality, muscle soreness, stress, mood), global recovery ratings, and—where available—biochemical markers such as creatine kinase. To avoid clinical connotations, we refer to “post-match recovery status/well-being” rather than implying pathology. Likewise, “explosive” outcomes are described here as rapid force–power performance (e.g., countermovement–jump height/power, sprint and agility tests). Finally, we use “single-session/immediate (minutes–hours)” to describe short-term effects of one stimulation session, and “multi-session/training-embedded” to describe protocols repeated over days to weeks and paired with practice or conditioning.

This scoping review aimed to map and synthesize clinical-trial evidence on tDCS in soccer players and to determine where the soccer-specific evidence indicates promise versus where claims remain tentative. Specifically, we addressed three questions: (1) What targets, montages, and application schedules (post-match window, pre-task single-session, and training-embedded multi-session) have been tested in soccer players? (2) Which outcome families—post-match recovery status/well-being, neuromuscular performance and adaptation, perceptual–cognitive/decision outcomes, and safety/tolerability—show the most consistent benefits, and under what conditions (target × task × timing)? (3) What are the main evidence gaps (e.g., power, standardization, women/elite representation, mechanistic readouts) that should guide future soccer-specific trials?

## 2. Methods

This scoping review examined how—and under what conditions—tDCS has been tested to influence soccer-relevant outcomes in healthy soccer players. To match the review questions, we grouped trials by application context: (i) post-match/post-exercise recovery-window protocols (typically within ~12–48 h after match load), (ii) single-session, pre-task protocols assessing immediate effects on performance or cognition (minutes–hours), and (iii) multi-session, training-embedded protocols intended to augment adaptation over days to weeks. Outcomes were synthesized within four families: post-match recovery status/well-being, neuromuscular performance/adaptation (strength, jump/sprint/agility, endurance, EMG), perceptual–cognitive/decision outcomes, and safety/tolerability. Because this is a scoping review, we did not fully follow every PRISMA item (Table S1).

### 2.1. Data Sources and Search Strategy

An electronic search was conducted in August 2025 to identify studies evaluating tDCS in soccer/football players by J.Ch. and D.K. We selected databases to maximize coverage across biomedical, sport-science, and trial literature: PubMed/MEDLINE for biomedical indexing and MeSH mapping; Scopus for broad multidisciplinary coverage (including sport and engineering journals); the Cochrane Library for controlled trials and systematic indexing of intervention studies; and Google Scholar plus ResearchGate to increase sensitivity for sport-science outputs and grey/early-access materials that may not yet be comprehensively indexed in traditional databases. Searches were limited to January 2008 through August 2025, reflecting the period in which modern tDCS protocols became widely used in the sport-performance literature. The core concepts were (i) soccer/football and (ii) tDCS, expanded with database-specific controlled vocabulary and synonyms (e.g., ”transcranial direct current stimulation”, ”tDCS”, and spelling variants), and combined using Boolean operators. In addition to database searching, we screened the reference lists of included studies and relevant reviews to identify any eligible trials missed by the electronic search. All retrieved records were collated and de-duplicated prior to screening using a combination of automated and manual checks.

### 2.2. Study Selection Criteria

We included interventional clinical trials published from January 2008 through August 2025 that evaluated tDCS in soccer/football players of any competitive level (youth to adult; amateur to elite). Eligible studies applied tDCS as the primary intervention (any montage, intensity, duration, or schedule), either alone or combined with training/recovery protocols, and reported at least one soccer-relevant outcome (e.g., physical performance, neuromuscular function, endurance, technical skill performance, perceptual–cognitive or decision outcomes, recovery/well-being indices, physiological/autonomic markers, or adverse events/tolerability). We included randomized and non-randomized controlled trials as well as crossover trials; language was not restricted when sufficient methodological and outcome data could be extracted.

We excluded (i) reviews, meta-analyses, commentaries, editorials, protocols, theses without extractable trial data, and case reports; (ii) observational studies without an interventional tDCS component; (iii) studies in non-soccer athletes or mixed-sport samples that did not report soccer-specific results separately; (iv) studies that used other non-invasive brain stimulation modalities without tDCS (e.g., rTMS, tACS) or combined neuromodulation approaches where the independent effect of tDCS could not be determined; (v) studies conducted in clinical populations where the primary aim was treatment of disease rather than soccer performance/recovery (unless the sample comprised soccer players and outcomes were soccer-relevant); and (vi) records lacking sufficient methodological detail or outcome reporting to confirm eligibility.

### 2.3. Screening Process

We used a multi-stage screening workflow to include relevant studies and exclude those not meeting prespecified criteria. Two independent reviewers (J.Ch. and D.K.) screened titles and abstracts.

#### 2.3.1. Title and Abstract Screening

At the first stage, each reviewer independently assessed titles and abstracts against the inclusion criteria, focusing on studies testing tDCS in football players.

#### 2.3.2. Full-Text Assessment

Records that passed the initial screen underwent full-text review to confirm that they were clinical trials, published between January 2008 and August 2025, irrespective of language, and were compliant with all eligibility requirements.

### 2.4. Data Extraction and Synthesis

We extracted (i) sample size and participant level/sex, (ii) stimulation target/montage, intensity, duration, and number/timing of sessions, (iii) comparator and blinding, and (iv) all soccer-relevant outcomes reported (subjective recovery status/well-being, neuromuscular performance/adaptation, perceptual–cognitive/decision outcomes, autonomic/physiological markers, and adverse events). Because included trials were heterogeneous in protocols, timing, and outcome measures, and were typically small, quantitative pooling (meta-analysis) was not appropriate. We therefore used a structured narrative synthesis and grouped findings by outcome family and application context (post-match recovery window, single-session pre-task, training-embedded multi-session). To avoid over-interpretation, we describe evidence as (a) more consistent when ≥2 independent studies using similar targets/timing showed effects in the same direction, and (b) tentative when findings relied on single studies, small samples, or non-overlapping outcome measures.

## 3. Results

**Figure 1:**
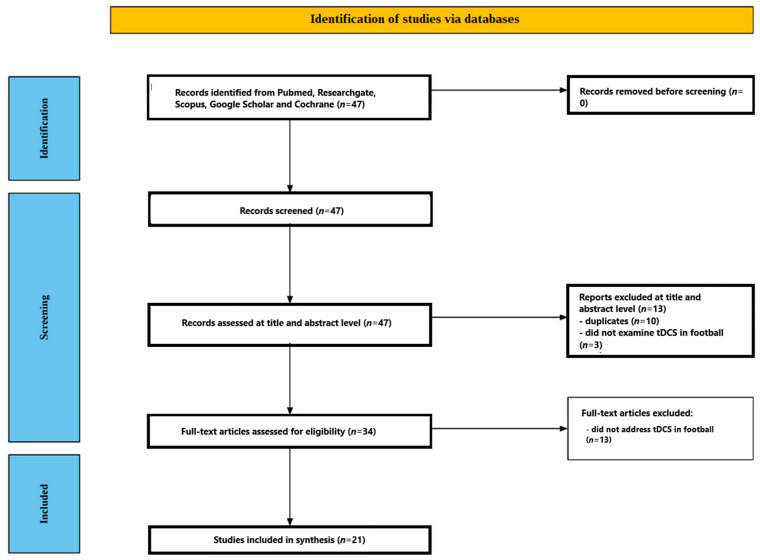
Flow chart depicting the different phases of the systematic review.

Figure 1 summarizes the study selection. We identified 47 records from the databases. After screening titles and abstracts, 13 were removed—10 were duplicates and three did not examine tDCS in a football context. We assessed the remaining 34 full texts and excluded 13 that did not evaluate tDCS effects on football performance. In total, 21 studies met the inclusion criteria and were included in the review [55,56,57,58,59,60,61,62,63,64,65,66,67,68,69,70,71,72,73,74,75]. The studies included in the review are presented in Table 1. Table 1 is intended as a descriptive map of the evidence (protocols, timing, outcomes, and design), not as proof of definitive effectiveness. Most trials were small (median sample size 24; range 12–66) and used heterogeneous measures of “functional status” (subjective well-being/recovery scales, technical/decision tasks, neuromuscular tests, autonomic indices). Therefore, we did not infer generalizable efficacy from single studies; instead, we summarize the direction and consistency of effects within outcome families and highlight where replication and standardized endpoints are currently insufficient.

### 3.1. Participants’ Characteristics

Across 21 studies (total *N* = 593), samples ranged from adolescent to adult soccer athletes, spanning world-class professionals to amateurs. The median sample size per study was 24 (12–66). Most participants were adolescents/young adults competing at U15–U23, university/college, or early-senior levels; the overall age span across studies was 14–40 years [55,56,57,58,59,60,61,62,63,64,65,66,67,68,69,70,71,72,73,74,75]. Sex distribution was predominantly male (≈76%, *n* = 450); females comprised ≈12% (*n* = 70), and three studies did not report sex (*n* = 73). Only two trials were female-only—elite professionals from a Brazilian first-division team (*n* = 13) and adolescent regional/national players (*n* = 20) [55,61]; three studies enrolled mixed-sex cohorts [76,77,78], while the remainder were male-only [57,58,59,60,62,63,66,67,68,69,70,72,74].

Competitive level and context were heterogeneous. Professional cohorts included world-class women [55], elite U-20 men from top Brazilian clubs [57], professional male outfield players [67], and first-division Brazilian men in a real-world recovery program [68]. Sub-elite/collegiate and university squads were common [58,59,60,62,63,70,71,72,74], alongside national-level players [64,69,73]. One study contrasted experts with novices within the same protocol [73]. Geographic representation was broad but concentrated in South America (especially Brazil) and East/Southeast Asia (China, South Korea, Malaysia), with additional samples from Iran/Kurdistan [56,57,58,59,60,62,63,64,66,67,68,69,70,71,72,73,74].

Training exposure was typical for the competitive tier studied: elite squads reported ~5–6 weekly sessions plus 1–2 matches [58], with the world-class women training 10–12 sessions per week [55]; amateur/college samples trained ≥2 sessions weekly and maintained active lifestyles [60,71]. Where specified, goalkeepers were excluded, or only outfield players were enrolled [64,67].

Inclusion/exclusion criteria were broadly consistent with sports-science trials: participants were generally injury-free and without neurological/psychiatric conditions or implanted devices [60,62,63,70,71,72,74,75]. Several studies targeted specific subgroups or conditions, including dynamic knee valgus [74], acute anxiety symptoms [75], and predefined match-exposure thresholds before recovery protocols [55,57,67,68]. Overall, the evidence base primarily reflects healthy, trained adolescent/young-adult soccer athletes, with limited female representation and few mixed-sex datasets.

Adult and late-adolescent male cohorts (e.g., U20 players, university/college players, third-division professionals, national-level athletes) typically clustered around late teens to mid-20s, with heights commonly between ~1.74 and 1.81 m and body masses roughly ~69 to 79 kg. Where reported, body mass index (BMI) in these male samples generally fell in the low-to-mid-20s (≈22–24 kg/m^2^), consistent with well-trained athletic populations. More detailed body composition data were less consistently provided, but when available, suggested substantial variability by age and training status: for example, a U20 cohort reported low body fat (~8–9%), whereas other trained adult samples reported moderate values (e.g., ~14% in professional outfield players assessed repeatedly during recovery).

Younger cohorts showed expected reductions in absolute size. Under-15 male players were smaller on average (mid-160 cm range, ~58 kg) yet still reported BMIs in the low 20s, alongside comparatively higher body fat (~20%), consistent with adolescent maturation. Female participants were represented in fewer studies but covered both elite and teenage groups. World-class professional female players averaged ~26 years of age with a mean height of ~1.66 m and a body mass of ~61 kg, while adolescent female players (15–17 years) were similar in stature (~1.67 m), with a body mass of ~60 kg and a BMI of around ~21 kg/m^2^. Several studies included mixed-sex samples (with women representing a minority), and reported training history and volume more consistently than full anthropometry; in these cases, sex distribution was documented, but height, body mass, BMI, or body composition were not always fully described. Overall, the anthropometric profile across studies reflects the sport’s broad developmental pipeline—adolescent to adult—while most adult male samples converged on a relatively narrow range of height, mass, and BMI, with body fat reported sporadically and differing by age group and competitive level.

### 3.2. tDCS Protocols

Across studies, stimulation parameters clustered around 2.0 mA for ~20 min delivered with 10–20 EEG montages tailored to the target outcome. Recovery and cognition-oriented trials predominantly used a bi-hemispheric prefrontal montage with the anode over the left DLPFC and the cathode over the right DLPFC (+F3/−F4) [55,57,67,75], with variants that placed the return electrode extracephalically (e.g., right shoulder) to limit contralateral cortical effects [59]. Some protocols applied dual-site anodal DLPFC (F3 and F4) with extracephalic cathodes in fatigue paradigms [64], and one program contrasted left-anodal/right-cathodal versus the reverse polarity to probe hemispheric asymmetries in decision-making [73]. Performance-focused work typically targeted the sensorimotor system: unilateral M1 using +C3/−Fp2 (or +C4/−Fp1) for limb-specific effects [61,66,74], midline +Cz with an inion cathode to drive bilateral M1 [58], or dual-site anodal M1 at C3 and C4 with shoulder cathodes during fatigue [64]. After sport-induced fatigue, a premotor approach placed the anode over the left PMd [65]. Several training and conditioning studies employed field-ready headsets (Halo Sport) that position electrodes across C3/C4/Cz with device-specific returns (often C5/C6), enabling deployment during or adjacent to practice [56,62,63,69,70,71].

Dose and timing were conservative and consistent: intensity was almost universally 2.0 mA (Halo reports ~1.98 mA) [56], with exceptions at 1.5 mA over M1 in a six-day pre-training protocol [66]. Session duration was usually 20 min, with shorter 10 min bouts in fatigue studies [64,65] and 15 min applications in acute performance or multi-week cognitive programs [58,59,66]; some conditioning blocks embedded 30 min sessions during training cycles [70,71]. When sponge electrodes were specified, pads were commonly 5 × 7 cm (35 cm^2^), yielding typical current densities of ~0.057–0.071 mA·cm^−2^ at 2 mA [55,61,62,63]. Scheduling mapped to the study aim: post-match recovery protocols delivered stimulation ~12–16 h after official matches or on the day after competition before follow-up assessments [55,57,67,68]; acute performance paradigms administered tDCS immediately before strength, jump, penalty-kick, or computerized cognitive tasks, with repeated assessments during and up to 60 min after stimulation [58,61,64,66,69]; and skill/conditioning studies paired stimulation with visuomotor drills or multi-week programs (core stability, lumbar stability, plyometrics, HIIT, sling training), typically 2–5 sessions per week over ~8 weeks [59,60,62,63,70,71,74]. Session counts spanned single-visit or 1–4-visit crossover experiments [55,56,57,58,61,64,65,66,69], three sessions within 48 h post-simulation [67], five sessions across one week [60], and up to 40 sessions over eight weeks for perceptual-cognitive training [59]; a club-embedded program layered tDCS onto routine recovery across a full season [68].

Sham control and masking were rigorous and largely standardized. Sham typically replicated montage and ramping but terminated current after ~30 s (often with 20–30 s ramps) [55,56,57,58,60,61,62,63,66,67,70,71,74,75]. Most studies were double-blind crossover; one M1 trial was triple-blind [58], headset work was sometimes single-blind [56], and the real-world comparison lacked randomization by design [68]. Where checked, blinding was adequate (e.g., ~29% correct condition guessing) [57]. Devices included conventional battery-powered DC stimulators with saline-soaked sponges [55,59,60,61,66,67,74,75], NeuroConn research systems [58], Starstim 8 for dual-site protocols [64], and Halo Sport for field deployment [56,66,67,69,70,71]. Ramping procedures (≈30 s up/down) minimized sensations; tolerability was high, with only mild itching/tingling/warmth reported and no serious adverse events across trials [55,56,57,58,60,61,62,63,65,66,67,68,69,70,71,74,75]. In aggregate, the literature converges on pragmatic, athlete-friendly implementations—~2 mA for ~20 min with +F3/−F4 for recovery/cognition and C3/C4/Cz for motor outcomes—delivered at moments aligned with the targeted adaptation (post-match, pre-task, or during training), under robust sham/blinding procedures and with excellent tolerability.

### 3.3. Outcomes

#### 3.3.1. Subjective Recovery and Well-Being

Subjective recovery was assessed primarily with the Well-Being Questionnaire (WBQ)—a 5-domain, 5-point instrument summed to an overall score (fatigue, sleep quality, muscle soreness, stress, mood)—and the Total Quality Recovery scale (TQR; 6–20), with some studies also using Subjective Recovery Scale (SRS)-type single indices. In world-class professional women tested 14–16 h post-match, both active and sham sessions improved perceptions from pre- to post-intervention, but anodal DLPFC (+F3/−F4, 2 mA, 20 min) produced a greater WBQ gain than sham (active: significant effect, *d* = 1.02; sham: moderate, *d* = 0.53; group example values: WBQ 17.17 → 20.25). By contrast, TQR increased over time in both conditions without between-arm differences (e.g., tDCS 12.46 → 15.46), indicating parallel improvements in perceived recovery quality despite the WBQ advantage for active stimulation [55]. An independent elite U-20 male cohort studied ~12–13 h after competition replicated the time-related rise in WBQ after both anodal and sham DLPFC sessions, with no between-condition difference, reinforcing that short-term recovery perceptions improve across the first post-match day regardless of stimulation in that design [57].

When stimulation was repeated across recovery windows, effects were more marked. In professional men completing a three-visit, 0/24/48 h post-simulation protocol, WBQ increased selectively under active tDCS (+F3/−F4, 2 mA, 20 min). In contrast, TQR rose similarly under both arms, mirroring the pattern in world-class women but extending it across 48 h of follow-up [67]. In a real-world, season-over-season comparison from a Brazilian first-division club, layering DLPFC tDCS (2 mA, 20 min) onto the team’s standard pneumatic compression routine yielded consistently better day-2 post-match perceptual outcomes than compression alone: perceived recovery +12% (*p* = 0.008), sleep quality +7.5% (*p* = 0.029), and muscle soreness −64% (*p* < 0.0001); biochemical recovery (CK) also improved in parallel (see Section 3.3.4), but the perceptual gains underscore a central contribution of tDCS to how players feel during recovery in applied settings [68].

Not all subjective indices shifted with stimulation. In a triple-blinded M1 protocol bracketing countermovement-jump testing, SRS and related perceptual measures were unchanged between active, sham, and control despite careful masking, suggesting that motor-cortex targeting in that single-session context does not reliably alter global recovery impressions [58]. Finally, although not a post-match study, a randomized trial in players with elevated anxiety symptoms found no between-arm differences in anxiety reduction or executive-function indices across seven daily DLPFC sessions; both arms improved over time, and higher anxiety correlated with poorer inhibitory control, indicating generalized time/practice or expectancy effects rather than a specific perceptual benefit of stimulation in that cohort [75].

Taken together, the most consistent subjective benefits appear when left-dominant prefrontal stimulation is applied within the first 12–48 h after match play or embedded in club recovery routines: WBQ improves reliably, often more with active tDCS than sham, whereas TQR/SRS typically track time-dependent recovery similarly across arms. These patterns suggest that tDCS can elevate multidimensional well-being (fatigue/sleep/soreness/stress/mood composite) beyond natural recovery. In contrast, global “quality” ratings of recovery tend to rise with rest and standard therapies irrespective of stimulation in tightly controlled laboratory crossovers.

#### 3.3.2. Neuromuscular Strength, Power, Speed, and Endurance

Across the corpus, neuromuscular outcomes were quantified with maximal voluntary isometric contractions (MVIC), countermovement-jump (CMJ) height and power, linear sprint and change-of-direction tests, and endurance indices (VO_2max_, shuttle, and Yo-Yo performance), with several studies also reporting EMG-based activation as a mechanistic correlate. The most stringent acute performance trial—a triple-blinded, randomized, controlled comparison targeting M1 with +Cz/inion, 2 mA for 15 min—found no between-group differences for CMJ height or power and no effects on heart rate, RPE, pain, or recovery perceptions, despite excellent masking and standardized warm-ups and testing sequences [58]. A youth cohort using a consumer headset (1.98 mA, 20 min to motor cortex at Cz with lateral returns) likewise showed no statistically significant changes in a broad fitness battery (flexibility, grip, back/leg dynamometry, standing long jump, vertical jump, 1 min sit-ups, 1 min push-ups) relative to sham. However, small favorable trends appeared for back/leg strength, long-jump distance, and sit-ups [56]. In contrast, an adolescent female sample receiving unilateral M1 anodal tDCS (2 mA, 20 min; anode over C3/C4, cathode supraorbital) exhibited clear, limb-specific strength gains: dominant-leg quadriceps MVIC rose +5.2% during stimulation, +6.3% at 30 min, and +9.4% at 60 min vs. baseline, whereas sham produced only −2.2% to +2.2% fluctuations; the non-dominant limb did not change, and no adverse events occurred (effects sizes small-to-large across time points) [61]. These single-session studies suggest that one tDCS session rarely enhances rapid force–power performance (e.g., CMJ height/power) in trained athletes. At the same time, isometric strength can improve transiently under targeted M1 stimulation, particularly in the dominant limb.

When stimulation was paired with training or repeated over days to weeks, benefits became larger and more transferable to functional outputs. In professional men studied after a simulated 90 min match, three recovery-window applications of +F3/−F4 (2 mA, 20 min at 0, 24, 48 h) elevated vastus-lateralis and rectus-femoris EMG amplitudes across the 48 h without altering CMJ height, implying neuromuscular facilitation that did not translate to gross jump performance in that context [67]. Multi-week motor programs consistently favored the tDCS arms. Combining lumbar-stability training with Halo-based stimulation over C3/C4/Cz for eight weeks (3×/week) increased trunk muscle activity and improved jump height meaningfully: countermovement jump rose 46.8 → 52.9 cm (hands on hips) and 53.3 → 61.1 cm (with arm swing), each outperforming exercise-only control under ANCOVA [62]. In university players with dynamic knee valgus, adding bilateral M1 anodal tDCS (2 mA, 15 min; C3/C4 with FP1 return) to an eight-week core program improved vertical-jump height by 25.3% (22.0 → 27.5 cm) versus 10.4% (21.0 → 23.1 cm) under sham, alongside superior knee alignment and agility (see §3.3.5 for biomechanics), with all group × time interactions statistically significant and large [74]. Sling-exercise paired with motor-cortex stimulation (2 mA, 30 min; twice weekly for eight weeks) yielded faster sprint and agility: 30 m sprint 4.51 → 4.37 s vs. 4.57 → 4.41 s in exercise-only, and *T*-test 11.82 → 11.11 s vs. 12.06 → 11.42 s; trunk EMG increases (erector spinae, rectus abdominis, external oblique) were also larger with tDCS [71]. In endurance-oriented conditioning, tDCS + HIIT (five sessions/week for eight weeks, 30 min stimulation during sessions) produced greater aerobic gains than HIIT alone: VO_2max_ 57.71 → 61.51 mL·kg^−1^·min^−1^ vs. 56.42 → 58.22, 20 m shuttle 113.34 → 121.51 reps vs. 112.76 → 119.38, and Yo-Yo IR 1298.61 → 1696.59 m vs. 1278.87 → 1644.28 m, all statistically significant [70]. These training-embedded effects—spanning power (jump height), speed/agility (sprint, *T*-test), and endurance (VO_2max_, shuttle, Yo-Yo)—support a model in which sensorimotor-targeted tDCS augments adaptation when coupled with structured practice.

Two additional strands refine interpretation. First, not all motor endpoints respond equally, even within the same paradigm: post-simulation prefrontal stimulation increased quadriceps EMG but left CMJ unchanged [67], and acute M1 stimulation did not modify CMJ despite robust methodology [58], underscoring that stretch–shortening, rapid force–power tasks may be less amenable to single-dose neuromodulation than isometric force or training-mediated adaptations. Second, site and montage matter. Strength and speed/agility improvements across multi-week programs overwhelmingly used M1-centric placements (C3/C4/Cz with extracephalic or frontal returns) [62,70,71,74]. In contrast, DLPFC protocols were more tightly linked to recovery perceptions and skill execution rather than raw neuromuscular power (see Section 3.3.1 and Section 3.3.3) [55,59,67]. Finally, headset-based, single-session applications in youth remain null on aggregate fitness metrics [56], highlighting the importance of dose (repetition), task coupling, and athlete maturity/training status for realizing neuromuscular benefits. In summary, the weight of evidence indicates that acute tDCS seldom improves jump power but can transiently enhance isometric strength; repeated, M1-targeted stimulation paired with training reliably yields larger gains in strength, jump performance, sprint/agility, and aerobic capacity than training alone, with excellent tolerability across cohorts.

#### 3.3.3. Cognitive, Perceptual, and Decision-Making Outcomes

Across the included trials, cognitive and perceptual outcomes were probed with laboratory tasks of attention, inhibitory control, working memory, mental flexibility, and social decision-making, alongside sport-specific decision tests and visuomotor reaction-time paradigms. A recurring pattern is that effects are highly target-, task-, and context-dependent: aligning montage and timing with the putative neural substrate of the outcome (e.g., M1 for attentional control under physical fatigue or DLPFC for executive/strategic processes) produces the most apparent benefits. At the same time, generic or misaligned protocols yield null or counterproductive results.

Under experimentally induced sports fatigue, a double-blind, counterbalanced trial in 23 national-level players compared dual-site anodal stimulation over M1 (C3 + C4, 2 mA, 10 min; extracephalic cathodes), DLPFC (F3 + F4, 2 mA, 10 min), and sham before computerized testing. Attention/inhibitory control improved only after M1 stimulation, with higher accuracy in the incongruent condition of the Stroop Color-Word task relative to sham, whereas DLPFC stimulation did not enhance Stroop. In the same experiment, working memory (2-back) was unchanged by either montage. DLPFC stimulation shifted decision strategy toward greater risk, producing lower net scores on the Iowa Gambling Task in later blocks than sham; M1 did not affect gambling behavior [64]. Thus, in a fatigued state, M1 facilitation supported attentional control, whereas bilateral prefrontal excitation did not, and, if anything, encouraged risk-tolerant choices.

In post-match recovery contexts, repeated left-dominant DLPFC stimulation did not generalize to classic executive measures. In professional men tested immediately, 24 h, and 48 h after a 90 min match simulation, three sessions of +F3/−F4 (2 mA, 20 min) improved well-being and neuromuscular activation but left Stroop interference unchanged across time and condition, indicating no measurable effect on interference control in that paradigm [67]. Similarly, in a randomized, double-blind clinical trial targeting anxiety in 23 players over seven consecutive daily sessions (+F3/−F4, 2 mA, 20 min), there were no between-arm differences on Stroop or Trail Making Test (TMT) despite within-group improvements over time; an exploratory analysis showed an inverse correlation between anxiety severity and inhibitory control, consistent with attentional control theory, but not specifically attributable to stimulation [75].

Task coupling appears to be a critical moderator when the goal is to accelerate perceptual-motor speed. In a five-session, parallel-group trial in 30 amateur players, pairing anodal left DLPFC (F3, 2 mA, 20 min; Fp2 cathode) with visuomotor training during minutes 5–15 produced significantly greater reductions in choice reaction time (CRT) than sham + training in the trained rectus femoris and a non-trained triceps muscle, while cognitive transfer to TMT and Digit Span was absent [60]. In a longer, 8-week program (40 sessions; 2 mA, 15 min) in 23 third-division athletes with F3 anode and extracephalic (right-shoulder) cathode, DLPFC stimulation did not change decision accuracy in small-sided games (SSG) or on a laboratory screen-based task. Still, it reliably shortened decision-making response time (≈655 ms → 626 ms). It restructured visual search behavior toward an expert-like pattern—more fixations per second with shorter fixation durations—implying more efficient attentional sampling without altering correctness rates [59]. These studies suggest that prefrontal neuromodulation can speed the decision process and tune visual exploration when repeated and/or paired with relevant practice, even if categorical accuracy remains stable.

Accuracy itself appears to be expertise- and polarity-dependent. In a single-blind, sham-controlled study of 66 participants (33 expert, 33 novice), bilateral DLPFC with left-anodal/right-cathodal polarity (F3 anode, F4 cathode; 2 mA, 20 min) improved video-based soccer decision accuracy in experts only from ≈52.3% to ≈59.9% (Cohen’s *d* ≈ 0.64), whereas the reverse polarity and sham were null; novices showed no benefit under any condition [73]. These lateralized effects align with models assigning the left DLPFC a dominant role in deliberative, rule-based selection, and further indicate that domain knowledge gates responsiveness—experienced players can capitalize on transient executive facilitation. In contrast, novices’ broader, less specialized networks blunt the effect.

Converging evidence from a semi-experimental, pre/post design in 60 male players distributed across skilled, semi-skilled, and amateur strata extends this expertise interaction. After three sessions of anodal prefrontal stimulation (2 mA, 20 min; anode over right DLPFC as described; contralateral supraorbital cathode), cognitive flexibility on the Wisconsin Card Sorting Test improved with a significant group effect (ANCOVA F = 36.65, *p* = 0.001, η^2^ = 0.391), driven by skilled and semi-skilled cohorts (both *p* < 0.001) but not amateurs (*p* = 0.422). In contrast, social decision-making on the Ultimatum Game improved across all expertise levels with a larger group effect (F = 70.86, *p* = 0.001, η^2^ = 0.554), suggesting that some prefrontal-dependent socio-evaluative processes are more universally modifiable than strategy-switching per se [72].

Finally, quasi-experimental evidence points to robust perceptual-motor speed gains when M1 is targeted repeatedly under ecological conditions. In 36 skilled male players randomized to anodal M1 (C3 anode/Fp2 cathode, 1.5 mA, 15 min × 6 days), sham, or control, the tDCS group showed a significant reduction in laboratory reaction time (354.25 ± 71.58 ms → 256.41 ± 63.72 ms; paired-*t*(11) = 18.703, *p* < 0.001) and a concurrent increase in match passing accuracy (46.00 ± 6.14% → 68.41 ± 5.83%; paired-*t*(11) = −20.178, *p* < 0.001), while sham and control remained stable; ANCOVA confirmed strong group effects (RT F = 66.521, *p* < 0.001, η^2^ = 0.811; performance F = 143.805, *p* < 0.001, η^2^ = 0.903) [66]. Although the non-randomized elements and sport-context confounds warrant caution, the magnitude and consistency of these changes reinforce that repeated M1 stimulation can translate faster sensorimotor processing into on-field technical execution.

In synthesis, the cognitive and perceptual literature indicates that single-session effects are most reliable when the target aligns with the proximal computation (e.g., M1 → Stroop under fatigue). In contrast, working memory shows minor sensitivity to brief tDCS in athletes. Repeated or task-coupled prefrontal stimulation can speed decisions and sharpen visual search without necessarily altering accuracy. In contrast, accuracy improvements emerge in experts under left-dominant DLPFC polarity (and may reverse toward risk-taking under bilateral anodal DLPFC in fatigue). These findings favor a specificity model: meaningful cognitive/perceptual benefits arise when montage, polarity, dose/schedule, and behavioral context are tuned to the targeted function and the athlete’s expertise level.

#### 3.3.4. Autonomic and Biochemical Markers

Autonomic regulation was evaluated chiefly via heart-rate responses during standardized submaximal exercise and early post-exercise recovery. At the same time, biochemical status was tracked with creatine kinase (CK) in the only season-long applied dataset. In an elite U-20 cohort tested the morning after competition, players completed a fixed-speed submaximal running test (10 km·h^−1^) immediately before and after either anodal or sham DLPFC stimulation; heart rate during exercise (HRex; last 30 s of the bout) was unchanged by time or condition, whereas heart-rate recovery at 60 s (HRR60) improved over time (*p* = 0.014) in both arms and the HRR index (HRex − HR at 60 s) likewise increased (*p* = 0.045) with no between-condition differences, indicating a time-dependent normalization of vagal reactivation rather than a specific tDCS effect in that design [57]. Converging evidence comes from a triple-blinded M1 study bracketing countermovement-jump testing: resting and post-exercise heart rate did not differ across active, sham, and control sessions, again suggesting that acute stimulation does not meaningfully alter cardiac kinetics under these conditions [58]. Where autonomic and metabolic load were used to induce (rather than modulate) fatigue, stimulation likewise did not change the load achieved: in a dual-site experiment that pushed athletes to exhaustion before cognitive testing, fatigue confirmation relied on canonical thresholds (heart rate near age-predicted maximum, RPE ≥ 17, blood lactate > 8 mmol·L^−1^); these markers served solely as gatekeepers and were not different across M1, DLPFC, or sham sessions [64]. A second fatigue model that quantified the stimulus more explicitly reported no between-session differences in riding duration, heart rate, RPE, or blood lactate during the fatigue bout preceding tDCS and task batteries (anodal vs. sham: RD 49.7 ± 8.3 vs. 50.2 ± 6.5 min; HR 174.1 ± 11.0 vs. 173.7 ± 14.9 bpm; RPE 19.8 ± 0.3 vs. 20.0 ± 0.0; Bla 8.7 ± 2.6 vs. 8.9 ± 4.2 mmol·L^−1^), reinforcing that the internal load attained was equivalent regardless of stimulation and that subsequent performance or perceptual changes cannot be attributed to differences in autonomic or metabolic stress at induction [65].

Biochemical recovery was addressed directly in a real-world, season-over-season analysis from a Brazilian first-division club that layered 20 min of DLPFC tDCS (2 mA; +F3/−F4) onto the squad’s existing day-after pneumatic-compression routine. On post-match day 2, the combined strategy was associated with a 76% reduction in CK concentrations (*p* = 0.001) relative to the prior season’s compression-only protocol, alongside perceptual gains in recovery (+12%) and sleep (+7.5%) and a 64% reduction in soreness (all *p* ≤ 0.029); notably, between-player variability in CK also narrowed, suggesting a more uniform physiological response to match load when tDCS was added [68]. While this applied dataset cannot isolate the central contribution of tDCS from contextual factors inherent to different seasons, the magnitude and consistency of the CK change—paired with unchanged training room practices aside from stimulation—support a biologically meaningful improvement in post-match muscle damage indices when a prefrontal protocol is used as an adjunct.

The autonomic findings across randomized, crossover designs indicate that single-session tDCS does not reliably modify heart-rate kinetics during fixed-intensity exercise or the first minute of recovery beyond the natural time-course observed across repeated post-match assessments. By contrast, in an applied, multi-match setting, adding prefrontal stimulation to standard peripheral recovery produced a significant, clinically relevant reduction in CK with tighter inter-individual dispersion, aligning the biochemical profile with the concurrent improvements in subjective status. These data suggest that while cardiac-autonomic markers (HRex, HRR60, HRR index) are predominantly time- and protocol-driven in tightly controlled single-session experiments, biochemical restoration may be more amenable to modulation when tDCS is integrated into comprehensive recovery routines rather than deployed as a single-dose laboratory intervention.

#### 3.3.5. Motor Control, Balance, and Biomechanics

Evidence for tDCS-driven changes in movement organization derives from EMG-based indices of muscle activation, center-of-pressure (COP) metrics of postural control, kinematic alignment during landing, and synergy-level coordination analyses during skill execution. In professional men undergoing a 90 min match simulation followed by three recovery-window sessions of left-dominant prefrontal stimulation (+F3/−F4, 2 mA, 20 min at 0/24/48 h), quadriceps EMG amplitudes were consistently higher with active tDCS than sham despite unchanged jump performance: vastus lateralis rose from ~11.7% MVC at baseline to 17.4% MVC at 24 h under anodal vs. 9.1% → 12.2% under sham, then declined by 48 h to 9.5% vs. 7.5%; rectus femoris tracked a flatter time course but remained elevated in the anodal arm (22.5% → 23.2% → 20.9% vs. 19.5% → 18.7% → 18.6%), yielding main effects of condition (higher activation with tDCS) and time (VL peak at 24 h) without interaction [67]. Two eight-week stability-centric programs showed that pairing sensorimotor-targeted stimulation with trunk conditioning augments both neural drive and function: lumbar-stability exercise plus Halo-based stimulation over C3/C4/Cz produced larger increases in erector spinae (31.3 → 40.6 μV), rectus abdominis (24.6 → 35.9 μV), and external oblique (27.7 → 36.3 μV) activity alongside greater gains in countermovement-jump height (hands on hips 46.8 → 52.9 cm; with arm swing 53.3 → 61.1 cm) than exercise alone [62], and sling-exercise plus tDCS similarly amplified trunk EMG (erector spinae 34.22 → 42.64 μV; rectus abdominis 26.17 → 36.24 μV; external oblique 28.74 → 38.07 μV) while improving 30 m sprint (4.51 → 4.37 s vs. 4.57 → 4.41 s in controls) and *T*-test agility (11.82 → 11.11 s vs. 12.06 → 11.42 s) more than sling alone [79]. Lower-limb activation and postural control also benefited when tDCS was layered onto plyometrics: in university men, rectus femoris (%MVIC) increased 27.12 → 33.64 and biceps femoris 20.33 → 26.41 with stimulation versus smaller gains in action-observation comparators, while static balance improved via reduced COP sway area (26.20 → 23.41 mm^2^) and path length (72.83 → 66.34 cm) and an increased limit of stability (904.54 → 967.64 cm^2^), all favoring the tDCS group on ANCOVA [63]. From a biomechanical-injury perspective, bilateral M1 anodal stimulation (C3/C4, 2 mA, 15 min) preceding core stability training in male players with dynamic knee valgus yielded superior frontal-plane alignment during single-leg landings (FPPA 166.5° → 175.9°, +5.65% vs. +2.26% under sham) with concomitant performance gains (vertical jump +25.3% vs. +10.4%; 8-hop time −21.05% vs. −14.27%), and all group × time interactions were significant with large effects [74]. At the coordinative level of skill execution, a laboratory penalty-kick model (20 national first-class males) revealed post-stimulation reorganization of neuromuscular control: spinal motor-pool output in L4–S1 activated earlier and more strongly (notably S1 and L4 amplitudes), an additional muscle synergy emerged (seven → eight), synergy activations narrowed temporally (more concentrated bursts), and anticipatory adjustments occurred sooner before support-foot contact and ball strike; co-activation indices shifted modestly (thigh RF–BF slightly down; shank TA–GAS slightly up), consistent with refined joint stabilization, although shot-outcome metrics were not the primary endpoints [69]. Together, these findings indicate that while explosive output (e.g., CMJ) may not acutely change under isolated sessions, tDCS can (i) elevate task-relevant EMG during recovery windows, (ii) enhance trunk and lower-limb activation and postural control when combined with targeted conditioning, (iii) improve frontal-plane knee mechanics in high-risk movers, and (iv) restructure phase-specific muscle coordination toward more efficient, temporally focused synergies during complex soccer actions.

#### 3.3.6. Safety, Tolerability, and Blinding Integrity

Across trials, adverse events were mild and transient (itching/tingling/warmth); no serious events were reported [55,56,57,58,60,61,62,63,65,66,67,68,69,70,71,74,75]. Sham integrity was generally good (e.g., ~29% correct guesses) [57]. Notably, one fatigue study observed risk-tolerant decisions after bilateral DLPFC anodal stimulation in the Iowa Gambling Task, underscoring the need for task-specific targeting [64].

### 3.4. Subgroup Analyses

Across the included soccer studies, the relationship among stimulation parameters, participant attributes, and outcome measures shows a coherent pattern. First, outcomes differ systematically by target region and montage. When stimulation is delivered over the dorsolateral prefrontal cortex (DLPFC)—most commonly with a bilateral prefrontal montage (+F3/−F4 or variants with extracephalic return)—the outcomes that most reliably move are those related to subjective well-being and soccer-relevant perceptual–cognitive function. In contrast, global recovery ratings and gross neuromuscular power outcomes are less consistent. In elite or professional recovery contexts, DLPFC stimulation is repeatedly linked to improved well-being questionnaire domains (fatigue/soreness/stress/mood/sleep components) relative to sham in some protocols (e.g., [55,67]. Yet, Total Quality Recovery (TQR) often improves similarly in active and sham conditions, yielding no clear between-condition separation (e.g., [55,67]). Likewise, when autonomic recovery is operationalized via heart-rate indices in elite post-match paradigms, improvements may occur over time but do not clearly differ between active and sham DLPFC stimulation [57].

In contrast, DLPFC stimulation appears more promising when the outcome is closer to soccer’s cognitive–technical demands: in professional players recovering from a soccer-match simulation, repeated DLPFC sessions enhanced soccer-specific technical performance on the Loughborough Soccer Passing Test, improving both speed and accuracy relative to sham, even though CMJ performance and Stroop interference were unchanged [67]. In competitive athletes receiving repeated DLPFC sessions during a training period, perceptual–cognitive efficiency also shifts in a way consistent with improved information processing, with faster decision-response times and more efficient visual-search metrics (more frequent, shorter fixations) despite limited change in decision accuracy per se [59]. At the same time, DLPFC effects are not uniformly beneficial across cognitive constructs. Under experimentally induced fatigue, dual-site DLPFC stimulation can bias decision-making toward riskier behavior on the Iowa Gambling Task relative to sham [64], highlighting that stimulation parameters and task demands can interact to produce directionally unfavorable shifts in risk preference rather than broad cognitive enhancement.

A second consistent moderator is participant expertise, particularly for DLPFC-driven decision outcomes. In video-based soccer decision-making paradigms, bilateral DLPFC stimulation improved decision accuracy only in expert players, and only under the specific polarity configuration that places anodal current over the left DLPFC with cathodal current over the right (L-anodal/R-cathodal); novices showed no benefit under any configuration [73]. This expertise dependence aligns with evidence from mixed-skill samples in which tDCS benefits for cognitive flexibility are confined to more experienced players. At the same time, simpler or more general social decision-making measures may improve across skill strata [72]. Taken together, these findings indicate that DLPFC stimulation is most likely to yield measurable gains when the stimulated network is strongly and efficiently recruited by the performer—i.e., in expert athletes—and when the outcome measure is tightly coupled to prefrontal executive control demands relevant to soccer performance (choice quality, response efficiency, scanning strategy), rather than broad, non-specific cognitive batteries.

By contrast, stimulation protocols targeting primary motor cortex (M1) or “motor cortex” regions—often implemented via conventional C3/C4/Cz placements or Halo device montages—show their clearest signal not as acute boosts to complex performance tasks, but as amplifiers of training adaptation and modulators of neuromuscular/biomechanical endpoints, especially when delivered repeatedly and paired with structured training. Single-session M1 stimulation can increase isolated strength outcomes in some populations—most notably dominant-limb quadriceps MVIC in adolescent female players [61]—suggesting that high signal-to-noise, muscle-specific strength tests may be more sensitive to acute motor-cortex modulation than whole-body power tasks. However, across well-trained male squads, acute M1 stimulation frequently fails to improve CMJ height or power, and subjective exertion or pain measures remain essentially unchanged (e.g., [58]); even when EMG indices shift with stimulation during recovery, this does not necessarily translate into jump performance changes [67]. The pattern becomes more consistent when tDCS is embedded within multi-week conditioning or neuromuscular programs. When motor cortex stimulation is combined with lumbar or core stability training, sling stabilization, plyometric blocks, or HIIT, studies repeatedly report larger gains than exercise-only comparators across trunk muscle activation, balance indices, sprint/agility performance, aerobic capacity (VO_2max_ and field tests), and jump measures [62,63,70,71]. Importantly, when participant attributes include a defined biomechanical risk phenotype—such as young male players with dynamic knee valgus—adding an M1-targeting tDCS protocol to an otherwise identical core stability intervention yields larger improvements in knee kinematics (FPPA) and functional performance tests than sham stimulation, implying that stimulation may facilitate motor relearning and neuromuscular reorganization when there is a clear “deficit target” for adaptation [74]. Motor cortex stimulation also shows selective cognitive relevance under fatigue: dual-site M1 stimulation can improve attention (Stroop incongruent accuracy) during sports fatigue without materially affecting working memory measures such as 2-back, again suggesting domain-specific rather than global cognitive effects [64].

Dose and timing further clarify the parameter–attribute–outcome relationship. Single-session tDCS tends to show more apparent effects when outcomes are relatively constrained and physiologically proximal (e.g., MVIC, select reaction-time metrics). In contrast, multi-session stimulation is more likely to separate from sham on outcomes that depend on learning, adaptation, or recovery across days. This is especially evident for motor-cortex stimulation paired with repeated training exposures, where the intervention functions less as a one-time performance enhancer and more as a neuroplasticity adjunct that increases the slope of adaptation [62,63,70,71,74]. For DLPFC protocols, multi-session dosing similarly appears to strengthen the signal for recovery-related well-being and for soccer-specific technical readiness, as shown by stronger well-being improvements and better passing performance after repeated post-simulation sessions [67] compared with single-session post-match paradigms in which sham and active tDCS often improve similarly on recovery indices [57]. Timing relative to load also matters: DLPFC effects are most coherently expressed in post-match/post-simulation recovery windows as changes in subjective well-being and technical readiness, while M1 effects are most coherent when stimulation is delivered adjacent to training over weeks as changes in neuromuscular activation, biomechanics, and fitness adaptations. In post-fatigue cognitive testing, both regions can produce measurable but dissociable effects—M1 on selective attentional control and DLPFC on decision-risk tendencies—underscoring that the same stimulation can be beneficial or undesirable depending on the cognitive construct probed and the fatigue state [64].

Finally, outcome sensitivity itself behaves like a moderator: across studies, WBQ-type well-being composites are more likely to show DLPFC-linked improvement than TQR, which often rises with time and standard recovery routines regardless of stimulation; CMJ is frequently insensitive to both DLPFC and M1 modulation in well-trained samples even when EMG indices change; and broad cognitive batteries (working memory, generic executive tasks) commonly yield null between-group findings unless the task is sport-specific or the sample is expert. Conversely, outcomes that are either closely tied to neural control strategies (visual search, choice reaction time under training, selective attention under fatigue) or represent cumulative adaptations (aerobic capacity, balance, biomechanical alignment, trunk activation) show the most apparent separation between active and control conditions. In sum, the included soccer literature suggests that the evident efficacy of tDCS is best predicted by an interaction: (1) stimulation target and polarity, (2) whether stimulation is embedded in repeated sessions and paired with training or recovery, (3) athlete expertise and phenotype, and (4) the proximity of the chosen outcome to the stimulated neural system, with DLPFC preferentially linked to well-being and expert perceptual–cognitive/technical readiness outcomes, and M1 preferentially linked to training adaptation, neuromuscular activation, biomechanics, and specific strength or attention endpoints.

### 3.5. Vote-Counting Synthesis and Effect-Ratio Summary

**Figure 2:**
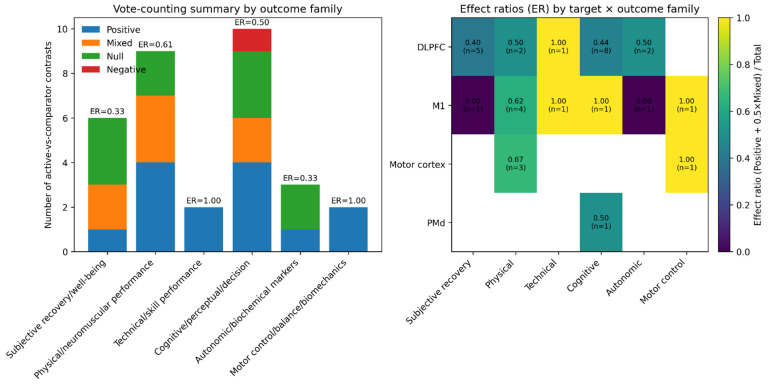
Vote-counting and effect-ratio summary across outcome families and stimulation targets. Left panel: stacked vote count of contrasts coded as Positive, Mixed, Null, or Negative. Right panel: effect ratios (ER = [Positive + 0.5 × Mixed]/Total) stratified by stimulation target (DLPFC, M1, motor-cortex headset placements, PMd). Coding is based on each study’s reported between-condition findings (Table 1 and Results narrative); uncontrolled pre–post designs were not included in ER calculations.

To complement narrative synthesis, we applied a vote-counting approach across active-vs-comparator contrasts and summarized trends using an effect ratio (ER = [Positive + 0.5 × Mixed]/Total). Across subjective recovery outcomes, effects favored active stimulation in 1/6 contrasts (ER = 0.33), with most studies showing null or mixed patterns. Physical/neuromuscular outcomes showed a higher proportion favoring stimulation (ER = 0.61), particularly in training-embedded multi-week paradigms. Cognitive/perceptual/decision outcomes were split (ER = 0.50) with one negative signal under bilateral prefrontal stimulation in a fatigue context. Autonomic/biochemical markers were mostly null in controlled acute designs (ER = 0.33), while motor-control/biomechanics outcomes favored stimulation in the limited available contrasts (ER = 1.00). These outcomes are presented in Figure 2.

### 3.6. Risk of Bias Assessment, Blinding Success Rate Assessment, Sample Size, and Statistical Power

The RoB-2 risk of bias assessment for randomized studies is presented in Table 2. The ROBINS-I risk of bias assessment for non-randomized studies is presented in Table 3. Furthermore, the blinding success rate, sample size description, and statistical power are assessed in each table.

### 3.7. Findings by Athlete Level Summary Table

The current findings assessed by athlete levels are presented in Table 4.

## 4. Discussion

The studies in this review show a heterogeneous but increasingly interpretable pattern: tDCS can modulate soccer-relevant outcomes, yet effects are contingent on what you target (brain region and construct), when you stimulate (fresh vs. fatigued; single vs. multi-session), and how you pair stimulation with practice or recovery. Broadly, we observed (i) positive results for some perceptual-cognitive skills (e.g., faster choices, more efficient visual search), technical execution under real or simulated match constraints, and several combined protocols (tDCS + training/recovery); (ii) mixed results for recovery perceptions and autonomic proxies, anxiety control, and countermovement power; and (iii) no effects in several pure performance tests (vertical jump, some HR/RPE endpoints, strength in trained athletes). These soccer findings align with the broader sports literature, where small-to-moderate improvements are most consistently seen in endurance-type and skill/decision tasks—especially under fatigue or when paired with practice. In contrast, maximal strength/power and precision skills in elite performers yield the most null results. Meta-analytic syntheses now mirror this split, reporting significant but modest, parameter-sensitive benefits, alongside pockets of non-replication and publication-bias concerns.

Importantly, the current soccer-specific literature is not yet sized or standardized to support firm efficacy claims: most studies include ≤30 players and employ diverse functional endpoints with variable reliability. Accordingly, our conclusions are framed as evidence patterns and practical hypotheses for future testing rather than definitive statements of effectiveness.

### 4.1. What tDCS Seems to Change in Soccer—And When

Two broad “families” of outcomes appear in the soccer set.

Affective/recovery and perceptual-cognitive control (well-being, perceived recovery, decision speed, anticipation, technical skill), where several studies reported advantages of left-DLPFC stimulation—acutely after match load or over short multi-session schedules [55,59,64,65,67,68,72,73]: These gains are consistent with non-soccer findings that anodal DLPFC tDCS can improve inhibitory control and extend endurance tolerance, plausibly via top-down regulation of effort perception and executive control [80,81]. In cyclists, left-DLPFC tDCS lengthened time-to-exhaustion while lowering RPE and HR, supporting a central (not peripheral) pathway [81]. Relatedly, under mental or sport-induced fatigue, prefrontal or orbitofrontal stimulation can preserve endurance or attention—an echo of the post-match/simulation recovery effects seen here [82]. The most coherent cross-domain explanation for prefrontal benefits links tDCS-induced shifts in executive control to the “psychobiological” model of endurance/skill regulation, in which perceived effort and inhibitory control constrain continuation and decision speed [83]. tDCS outcomes are consistent with a top-down route rather than peripheral facilitation. Relatedly, maintaining performance under mental or sport-induced fatigue aligns with evidence that fatigue impairs endurance chiefly by elevating perceived effort and taxing attention [84]. These convergences make prefrontal stimulation a principled target when tactical choices and self-regulation dominate outcomes.

Neuromuscular/physical outputs (strength, jump, sprint/agility, balance, aerobic fitness), where results ranged from clear positives when tDCS was paired with training [62,63,70,71,74] or delivered in focused lab tasks [60,61,66,69] to several nulls [56,58,75]: This split mirrors other sports: meta-analyses suggest small, heterogeneous, and task-specific effects, with the most reliable benefits in endurance-type or sustained tasks, and less consistent effects for maximal strength/precision skills [76,85]. Individual studies outside soccer likewise show that M1 or bilateral/extracephalic montages can raise endurance performance or vertical-jump metrics in some circumstances, yet other well-controlled trials—sometimes even with HD-tDCS—find no performance change [77,86,87].

### 4.2. Why Results Differ: Target × Task Specificity

Targeted cortex matters. Left-DLPFC stimulation aligned with improvements in well-being after load and with faster/more accurate technical decisions in soccer. This maps onto endurance-sport evidence that DLPFC tDCS can enhance inhibitory control and prolong effort tolerance—interpreted within psychobiological models where perceived effort governs stopping [80,81]. In contrast, M1 stimulation maps better onto force/endurance endpoints, sometimes increasing torque, delaying supraspinal fatigue, or modestly improving lower-limb explosive tasks in athletes or trained adults [88,89]. The few cerebellar and oPFC sport studies illustrate even tighter task–target coupling: cerebellar tDCS can improve shooting precision, and oPFC stimulation may help preserve endurance under mental fatigue—effects that make sense given each region’s role in timing/coordination and valuation/affective control, respectively [82,90].

Montage and current return also matter. Several positive endurance studies used bilateral M1 with extracephalic returns (anodes over motor cortices, cathodes on shoulders), which alters current flow relative to cephalic returns and is hypothesized to reduce perception of effort more effectively [87]. Soccer protocols varied widely (F3/F4; C3/C4; extracephalic; commercial multi-contact headsets), which likely contributed to variability—especially for strength/power where current density at the leg M1 “hotspot” is critical.

### 4.3. State-Dependence, Training Status, and Expertise

tDCS effects are state-dependent. Benefits in soccer were most consistent after fatiguing load (e.g., post-match and post-simulation: [55,57,67,68] or when paired with active practice [60,62,63,70,71,74]). This matches non-soccer observations that stimulation may be larger when the system is taxed (fatigue/mental fatigue) or when learning/updating occurs [82]. Expertise also modulates outcomes: in soccer, expert decision accuracy benefited from specific bilateral DLPFC polarity [73], while several novice or youth samples showed more minor or null effects [56,58]. Across other sports, effects sometimes scale with training status or are confined to experts, implying ceiling effects and distinct neural control strategies in novices [79].

### 4.4. Single- vs. Multi-Session and Pairing with Training

A recurring theme in soccer is that multi-session prefrontal stimulation during the 0–48 h recovery window [67] or repeated pairing with training (core/lumbar stability, sling, HIIT; [62,70,71,74] yielded clearer and broader transfer (EMG patterns, sprint/agility, VO2-linked tests) than single-shot sessions. This dovetails with broader sport studies and recent meta-analyses: acute effects are minor and protocol-sensitive; repeated exposure and concurrent practice tend to produce more durable or larger benefits [76,91].

### 4.5. Why Some Strong Nulls?

Taken together, the strongest null findings in our soccer dataset are not surprising when viewed through what is now a consistent picture emerging across sport tDCS: average effects are small-to-trivial, highly variable across people and protocols, and extremely sensitive to methodology. Recent quantitative syntheses in athletes and active populations estimate pooled acute effects around SMD ≈ 0.19–0.45, with apparent between-study heterogeneity and signals of small-study/publication bias [78]. Against that backdrop, several design choices in our included soccer studies make “true” effects easy to miss (type-II error) and apparent effects easy to dilute (placebo, carry-over, and measurement noise). Below, we outline the most plausible, evidence-based reasons for the strong nulls we observed.

#### 4.5.1. Outcome—Target Mismatch and Under-Engagement of the Leg Motor Representation

Several soccer trials used broad, conventional 5 × 7 cm pads over F3/F4 or Cz/inion to influence prefrontal control or M1. Finite-element and in-vivo work shows that standard montages produce diffuse, shallow fields and that individual head anatomy and pad placement dominate, meaning that gyri receive adequate field strength. The lower-limb M1 (medial wall) is particularly difficult to engage with conventional pads; even minor positioning errors or hair/impedance issues can shunt current and reduce dose at the interhemispheric fissure [92,93,94]. Thus, null changes in jump height or knee-extensor HR metrics after “M1” stimulation may reflect insufficient engagement of the leg motor representation rather than the absence of a physiological effect per se. Newer modeling-guided or HD-tDCS montages can increase focus, but superiority over conventional pads for whole-body performance remains unproven or modest [95].

#### 4.5.2. Dose and Timing: Non-Linearities, Carry-Over, and the “Train-with-Stimulation” Rule

tDCS shows non-linear dose–response characteristics (intensity × duration), with parameter ranges beyond ~20 min sometimes reversing or washing out expected excitability changes; after-effects can last tens of minutes to hours and accumulate across sessions [96,97]. Crossover designs common in sport can suffer short and long carry-over sunless washouts are conservative, and order is modeled appropriately. Methodological reviews warn that order/period effects are often under-handled in tDCS crossover trials [98]. In parallel, motor learning studies demonstrate that the most reliable behavioral benefits arise when training is performed during stimulation and repeated over days (online, multi-session), boosting consolidation rather than acute outputs measured immediately after a single “offline” dose [99,100]. Where our soccer studies administered one 10–20 min session after a match or before isolated tests, without concurrent high-challenge practice, nulls were common. In contrast, multi-session protocols paired with training are more promising in the broader sport literature.

#### 4.5.3. Placebo, Sham, and Blinding—Especially for Perceptual Outcomes

Perceived recovery (TQR), well-being, and RPE are highly placebo sensitive. Blinding with 2 mA stimulation is imperfect in many contexts, and conventional “brief-ramp” sham may not be biologically inert. This can equalize groups on subjective scales and inflate improvements in both arms [51,101]. At the same time, “end-of-study guess” is itself an unreliable blinding check, complicating interpretation when manipulation checks read as successful [102]. The net effect is that perceptual outcomes often improve over time with both active and sham—precisely what we observed in post-match recovery protocols—while harder physiological or performance endpoints remain unchanged.

#### 4.5.4. Statistical Power, Measurement Sensitivity, and the Most Minor Worthwhile Change

Meta-analyses repeatedly note small samples (often *n* ≈ 10–20) and heterogeneity in sport tDCS; underpowered studies plus trial-to-trial noise in field tests (e.g., CMJ, heart-rate recovery) make detecting minor actual effects unlikely. For example, the typical error for CMJ in trained athletes is ~1–2 cm, depending on the system; effects smaller than this are practically undetectable without large samples or repeated-measures reliability phases [103]. Reliability is acceptable for subjective scales (TQR/WBQ) but still vulnerable to context and instruction drift across congested schedules [104,105]. When the plausible actual effect is SMD  <  0.3, even well-run, double-masked crossovers will often read null.

#### 4.5.5. Inter-Individual Variability (“Responders”) Driven by Anatomy, Neurochemistry, Sex/Age, and Task

Half or more healthy participants can show minimal or paradoxical excitability modulation to canonical M1 anodal tDCS, and variability for the lower-limb representation appears at least as large [106,107,108]. Modeled electric fields explain a meaningful portion of this heterogeneity; differences in skull/CSF thickness, gyral geometry, and pad contact (hair, sweat, saline volume) shift both magnitude and orientation of fields at the target [94,109,110]. Athlete-specific factors—fatigue state, autonomic load, hormones, caffeine—further condition responsiveness via state-dependent mechanisms. In elite squads, this produces mixtures of positive, null, and even negative responders that average to “no effect.”

#### 4.5.6. Device and Montage Idiosyncrasies (e.g., Consumer Headsets)

Some included trials used commercial headsets (e.g., Halo) with fixed “primer” electrodes and default montages. Independent sport studies with these systems show mixed outcomes (from small endurance benefits to clear nulls), consistent with the broader literature’s minor average effects and high variance; moreover, the manufacturer is now defunct, underscoring the need to treat device-specific claims cautiously and to report impedance/contact quality rigorously [111,112,113].

#### 4.5.7. Task Choice: General Tests vs. Sport-Specific, Cognitively Loaded Skills

Systematic reviews increasingly highlight that tDCS effects are easier to elicit in sport-specific tasks under time pressure (complex skills, pacing, decision making) than in generic lab proxies [90]. Several of our strong nulls relied on brief, generic outputs (CMJ, handgrip/leg dynamometry) rather than soccer-specific skills under cognitive load; in contrast, outside soccer, positive studies commonly pair stimulation with demanding sport-relevant training (e.g., repeated-sprint cycling, skill acquisition blocks, or endurance pacing) [89].

#### 4.5.8. Training-Load Confounding and Ecology

In-season studies run within congested fixtures confront fluctuating loads, sleep, soreness, and nutrition—all of which move the same endpoints tDCS seeks to influence—and inflate within-subject variance. Even when match demands are “similar,” residuals from microcycle differences can dominate minor neuromodulatory effects, again biasing toward nulls in ecologically valid designs.

### 4.6. Conditions Under Which Efficacy Is Most Certain

Although the number of trials remains limited and protocols are heterogeneous, several patterns emerge regarding when efficacy is most likely. Across the included studies, benefits are most consistently observed when (i) tDCS is delivered acutely in temporal proximity to the tested task (pre- or online stimulation), (ii) stimulation is task-congruent with the targeted neural substrate (most commonly motor-related regions when outcomes are motor/technical), and (iii) outcomes are immediate, standardized, and sensitive to small performance changes. Conversely, when protocols vary widely in montage/intensity, when outcomes are distal or multifactorial (e.g., match performance proxies), or when assessment occurs days to weeks after stimulation without tight control of training load and context, efficacy becomes difficult to attribute and less reliable. These observations support prioritizing future trials that align stimulation timing and target a clearly defined soccer-specific performance endpoint, while maintaining strong sham controls and blinding.

## 5. Mechanisms of Action of tDCS in Improving Sports Performance

tDCS can acutely bias membrane potentials and trigger longer-lasting plasticity in circuits that govern effort perception, motor output, sensorimotor integration, decision-making, and autonomic regulation with appropriate dosing and timing. Below, we synthesize convergent evidence—spanning human neurophysiology, neuroimaging, pharmacology, and exercise science—to outline plausible pathways by which tDCS can support athletic performance.

### 5.1. Cortical Excitability and Synaptic Plasticity

At the most proximal level, anodal tDCS shifts the resting membrane potential of cortical pyramidal neurons toward depolarization (and cathodal toward hyperpolarization), thereby biasing spike probability during synaptic input without directly eliciting action potentials. In the human motor cortex, this manifests as polarity-specific, minutes-to-hours changes in cortico-spinal excitability indexed by TMS-evoked MEPs: anodal stimulation increases MEP amplitude, cathodal reduces it, with “online” effects during stimulation and “after-effects” that outlast the train. The magnitude and persistence of these shifts scale with current intensity and duration, such that longer anodal trains produce sustained excitability elevations. In contrast, brief trains exert only a transient influence [27,29,114].

Those after-effects are not simply passive “battery” phenomena; pharmacology shows they rely on synaptic plasticity machinery. Blocking NMDA receptors (e.g., dextromethorphan) abolishes the enduring post-stimulation changes for both anodal and cathodal tDCS, whereas facilitating NMDA transmission (e.g., D-cycloserine) prolongs or strengthens them. Additional work implicates voltage-gated sodium and calcium channels in shaping the direction and duration of the effect, consistent with Hebbian, LTP/LTD-like processes rather than mere membrane polarization [34,115].

Magnetic-resonance spectroscopy (MRS) provides a complementary neurochemical window: anodal M1 tDCS reduces local GABA concentration (and can modulate glutamatergic metabolites), creating a transiently disinhibited cortical state permissive for plasticity. Crucially, the size of the tDCS-induced GABA decreases correlates with individual gains in motor learning and the strength of task-related BOLD activity and connectivity—linking the chemistry to behaviorally meaningful change. These GABAergic shifts also couple to network-level reconfiguration (e.g., strengthened motor-network connectivity at rest), offering a plausible bridge from local synaptic modulation to large-scale performance effects [116,117,118].

Animal and cell-level work converge on a trophic substrate for these phenomena. Applying weak direct current over the sensorimotor cortex facilitates BDNF/TrkB-dependent synaptic potentiation; blocking this pathway prevents the augmentation of learning-related plasticity. This positions tDCS as a “plasticity primer” that biases ongoing training toward LTP, accelerating formation and consolidation of more efficient sensorimotor maps—a mechanism directly relevant when athletes pair stimulation with drills or conditioning [31].

Critically, the relationship between dose and effect is not linear. In the motor cortex, increasing intensity and/or duration modulates not only the size but sometimes the sign of the after-effect (e.g., strong/long cathodal can flip from inhibitory to excitatory), and interdependencies between current strength and train length set a finite window for stable, LTP-like changes. These non-linearities, together with individual anatomy, mean that the in-brain electric field (typically on the order of a few tenths of a volt per meter) varies across people and regions, helping explain heterogeneous behavioral outcomes unless montages and doses are tailored [94,119,120].

Finally, plasticity is state- and history-dependent. Repeating anodal tDCS within a specific interval can convert short-lived facilitatory effects into late, NMDA-dependent LTP-like plasticity (l-LTP), whereas delivering stimulation outside that window, or onto a cortex recently driven into high excitability, can blunt or even reverse the response (homeostatic metaplasticity). Practically, this argues for aligning stimulation with practice and recovery blocks to exploit permissive states—maximizing synaptic gain on task-relevant circuits while avoiding saturation [121].

### 5.2. Network-Level Reconfiguration: Frontoparietal, Motor, and Cerebellar Systems

tDCS does not act solely on the cortical patch beneath an electrode; it reshapes interactions among distributed networks that support attention, executive control, sensorimotor integration, and balance. Resting-state fMRI after prefrontal anodal stimulation shows reproducible, polarity-specific remapping within canonical networks: connectivity increases within the frontoparietal control network and between control and default-mode subsystems, alongside topology changes (e.g., altered clustering and path length), consistent with a shift toward more efficient global communication. These effects appear within minutes and persist beyond the stimulation train, indicating that tDCS nudges large-scale network operating points rather than producing a fleeting local gain [122].

Motor-system tDCS similarly reorganizes connectivity along cortico-subcortical loops. After anodal M1 stimulation, both EEG and fMRI reveal strengthened functional coupling among nodes of the cortico-striato-thalamo-cortical circuit that govern movement initiation, selection, and vigor. Graph-theoretic analyses demonstrate reweighting of interregional edges with increased integration of motor hubs during task and rest. At the same time, seed-based fMRI confirms modulation of cortico-striatal and thalamo-cortical links—mechanisms that map naturally onto improved corticomotor efficiency under load [123,124].

Beyond functional coupling, neuromodulatory signatures align with these network changes. PET with [11C]raclopride shows that bifrontal (DLPFC) tDCS increases endogenous dopamine release in the human striatum—an effect observable as reduced ligand binding potential. Because dopamine gates cortico-striatal plasticity and regulates motivation and effort discounting, this subcortical response provides a mechanistic bridge between prefrontal stimulation and shifts in decision vigor, exploration–exploitation balance, and sustained task engagement during training or competition. Convergent multimodal work indicates that tDCS can amplify activity within mesostriatal regions, underscoring a systems-level (cortex-to-basal ganglia) pathway rather than a purely local cortical phenomenon [125,126].

Network plasticity is state- and montage-dependent. Bilateral or high-definition (HD) montages that better focus the induced electric field alter resting-state connectivity differently than conventional sponge montages; individual variability in anatomy (and thus in-brain E-field strength and orientation) explains a substantial fraction of the heterogeneity in network responses. Real-time and “during-stimulation” fMRI further show that bilateral DLPFC tDCS can modulate network coupling while the current is on, with after-effects that evolve in the minutes following offset—evidence for dynamic reconfiguration rather than a static on/off switch. These observations argue for tailoring electrode geometry and dose to the targeted network and timing stimulation relative to practice to exploit permissive brain states [127,128].

Cerebellar tDCS adds another axis of network control. By modulating cerebello-thalamo-cortical excitability, anodal cerebellar stimulation can alter effective connectivity within cortico-striato-cerebellar learning loops and improve postural control and balance in behavioral studies. Imaging during cerebellar tDCS demonstrates task-specific shifts in cerebro-cerebellar coupling, consistent with enhanced internal-model prediction and error-based adaptation—the exact computations athletes rely on for stance stability, gait, and rapid recalibration under perturbation. While not every balance task benefits (likely reflecting task complexity and ceiling effects), meta-analytic and experimental evidence support cerebellar network engagement as a viable route to performance-relevant change [129,130,131,132].

In aggregate, these findings position tDCS as a network-level modulator: prefrontal stimulation tunes control–default interactions and frontostriatal gating (shaping attention, effort, and decision policy), motor-cortex stimulation strengthens cortico-basal-ganglia-thalamo loops (enhancing drive and selection), and cerebellar stimulation refines cerebello-cortical prediction–error cycles (improving balance and coordination). When delivered with the right montage and at the right time relative to practice or recovery, this coordinated reconfiguration supplies the systems-neuroscience substrate for durable, task-specific gains in athletic performance.

### 5.3. Effort Perception, Pain Modulation, and Autonomic Control

A central pathway by which tDCS can enhance athletic output is by re-tuning how the brain appraises how hard something feels (perceived exertion), how much it hurts (pain), and how the body is regulated (autonomic balance). In contemporary psychobiological models of endurance, the conscious sense of effort is generated by cortical control systems (not by muscle afferents per se) and gate-keeps pacing, persistence, and task termination. Key nodes include the dorsolateral prefrontal cortex (DLPFC) exerting top-down control over salience/interoceptive hubs (anterior insula, dorsal anterior cingulate), which integrate bodily signals with goals and motivation. By biasing excitability in these circuits—most commonly with anodal stimulation of left DLPFC or M1—tDCS can shift the internal “cost” calculus of continuing intense exercise [133,134].

The first is effort perception. Controlled endurance trials show that anodal left-DLPFC tDCS can prolong time-to-exhaustion while lowering rating of perceived exertion (RPE) at matched workloads, often with secondary signs of deeper physiological strain tolerance (e.g., higher end-exercise lactate, lower heart rate), consistent with a central re-scaling of effort rather than peripheral facilitation alone [81]. Meta-analytic work now indicates a small-to-moderate overall reduction in RPE with anodal tDCS, whether the target is DLPFC or M1, with standard Borg 6–20 and OMNI scales both sensitive to the change [49]. DLPFC up-regulation mechanizes inhibitory control and attentional allocation, making aversive interoceptive cues less behaviorally dominant during sustained efforts. Notably, montage and task matter: some studies find null effects when stimulation is suboptimally targeted or when self-paced strategies dampen central gating demands.

The second is pain modulation. tDCS engages canonical descending pain-control pathways, offering a second lever on performance. Across clinical and experimental paradigms, anodal M1 and DLPFC stimulation reduce pain intensity and pain affect, with multiple systematic reviews and guidelines now supporting short-term analgesic benefits. Molecular imaging provides convergent mechanisms: μ-opioid PET demonstrates that both placebo-like expectancy and real M1/DLPFC tDCS increase endogenous μ-opioid neurotransmission in periaqueductal gray, thalamus, and prefrontal cortex—regions pivotal for antinociception—while rTMS, working with naloxone blockade, triangulates an opioidergic contribution to stimulation-induced analgesia. These top-down effects likely act in parallel with attentional reappraisal (DLPFC) and sensorimotor gating (M1), together dampening exercise-induced pain and the affective “cost” of sustained high output [135,136,137,138].

The third is autonomic regulation and recovery. A complementary route to improved tolerance is a shift toward parasympathetic dominance—supporting calmer cardiorespiratory control during work and faster recovery between bouts. Prefrontal tDCS can acutely increase high-frequency heart-rate variability (HF-HRV; a vagal marker) and reduce stress-linked neuroendocrine responses, with a growing systematic review/meta-analytic base indicating overall improvements in HRV metrics at rest and under challenge. Dose, polarity, and individual electric-field strength appear to moderate effect sizes, consistent with state- and montage-dependence; nevertheless, even single sessions over DLPFC have attenuated autonomic arousal to psychosocial and inflammatory stressors, and patient studies report feasible modulation of cardiac autonomic indicators during acute illness. In athletic contexts, such shifts map onto lower perceived strain for a given output, more efficient pacing, and improved overnight recovery readiness [139,140,141,142].

### 5.4. Neuromuscular Drive and Motor-Unit Behavior

At the efferent end of the performance cascade, anodal stimulation over primary motor cortex (M1) can increase the descending neural drive to agonist pools and subtly retune the spinal interface through which that drive is expressed. In humans, the cleanest evidence that tDCS augments central drive during high-effort contractions comes from twitch interpolation/voluntary activation paradigms: after repeated sessions of M1 anodal tDCS, participants show higher cortical voluntary activation (i.e., a more minor superimposed twitch when a supramaximal doublet is delivered during an MVC) together with strength gains—direct readouts of improved corticospinal contribution to force. Genetic moderators such as BDNF Val66Met can scale these effects, underscoring their plastic origin rather than mere membrane bias [143].

Once descending command reaches the cord, the patterning of motor-unit recruitment and firing determines torque, rate of force rise, and steadiness. Several independent signals point to tDCS-sensitive adjustments here. First, V-waves—evoked during MVCs and widely interpreted as an index of supraspinal drive reaching the motoneuron pool—rise after anodal M1 stimulation, parallel with larger surface EMG amplitudes in the same muscles [144]. Second, submaximal H-reflex metrics (e.g., H normalized to M) can increase without changes in H-max, a signature consistent with a selective easing of inhibitory spinal interneuronal gating rather than a nonspecific motoneuronal “arousal.” These findings indicate that tDCS can amplify the efferent volley and bias spinal circuits to pass it more effectively when task demands are high [144].

A complementary window on drive is corticomuscular coherence (CMC)—the frequency-specific coupling between cortical rhythms and motor-unit activity measured with EEG–EMG [145]. High-definition M1 tDCS has increased CMC (notably in the beta range linked to steady force output). This implies tighter standard synaptic input from cortex to motoneurons and more efficient transmission along the corticospinal tract during voluntary actions [146]. Related oscillatory neuromodulation studies converge on the same interpretation: strengthening cortico-spinal coupling improves the fidelity of motor commands, a prerequisite for precise, economical movement under load [147,148].

These central and spinal adjustments express behaviorally as changes in force kinetics. In ballistic and explosive tasks, anodal M1 tDCS has increased peak rate of force development (pRFD) in upper- and lower-limb models—an outcome that, mechanistically, requires faster recruitment of higher-threshold units and/or higher instantaneous discharge rates early in contraction. Not all protocols replicate (particularly with suboptimal montage or insufficient current density), but across positive studies, the direction of effect aligns with the physiology above: more effective corticospinal drive and reduced inhibitory gating enable steeper force ramps [88,149].

### 5.5. Skill Learning, Consolidation, and Decision Processes

tDCS influences skill acquisition through a two-step cascade: it first biases the online encoding of new sensorimotor policies while practice occurs. Then, it amplifies offline consolidation so that performance measured at a later retest is higher than expected from practice alone. In humans, anodal M1 tDCS administered during multi-day training selectively enhances offline gains—i.e., improvements expressed hours to days after practice rather than within the same session—thereby accelerating total skill acquisition and prolonging retention. This pattern was demonstrated across five consecutive days of training with retests extending beyond 3 months, where anodal M1 stimulation increased total learning by boosting between-session improvements rather than within-session gains [99].

Neurochemically, these behavioral benefits align with a transient, learning-permissive shift in local inhibition–excitation balance. In vivo MRS shows that anodal M1 tDCS acutely reduces GABA; critically, the magnitude of this decreases scales with individual motor learning and with learning-related fMRI activity, linking a measurable cortical disinhibition to durable skill improvements. Subsequent work further ties the size of the tDCS-induced GABA change to the modeled electric-field strength within M1, helping explain inter-individual variability and underscoring the value of dose/montage tailoring in athletes. At the network level, the same manipulations strengthen functional connectivity within the motor resting-state network immediately after stimulation—plausibly facilitating consolidation by improving communication among nodes that encode the trained skill [118,150,151].

Different nodes contribute distinct “learning roles,” and tDCS can leverage these roles. Cerebellar anodal stimulation reliably speeds error-based adaptation (faster reduction of movement errors to a new sensorimotor mapping). In contrast, anodal M1 preferentially stabilizes what has been learned, improving retention after removing the perturbation. This dissociation—often summarized as “the cerebellum learns the model, M1 stores it”—maps neatly onto athletic contexts where rapid recalibration (e.g., to surface, ball, or fatigue changes) must be followed by robust retention of the new policy across sessions and matches [152].

Timing matters. When stimulation is paired with repetitive practice and followed by a consolidation window, gains are larger and more reliable than with stimulation at rest. Premotor-cortex (PMd) anodal tDCS during sequence practice, for example, enhances the overnight offline gain that typically appears only under optimal practice schedules, indicating that properly targeted stimulation can convert practice that typically yields modest consolidation into practice that benefits from sleep-dependent plasticity. More generally, multi-day stimulation-plus-practice protocols substantially consolidate fine force control and dexterous skills more than single-session applications [100,153,154].

Decision processes that gate skilled performance—selecting the right action at the right time, calibrating risk, and credit assignment from feedback—are also malleable. Bifrontal DLPFC tDCS has been shown with PET to increase endogenous striatal dopamine release, directly implicating frontostriatal neuromodulation in tDCS’s cognitive effects. Dopamine is a key teacher signal for reinforcement learning, vigor, and the exploration–exploitation trade-off; augmenting dopaminergic tone via prefrontal stimulation offers a mechanistic route to faster learning from feedback and greater decision efficiency during play. Converging behavioral data show that modulating DLPFC excitability can reduce risk-taking in laboratory paradigms (e.g., Balloon Analog Risk Task; gambling tasks), shifting choice policy toward more deliberate, reward-maximizing actions—precisely the kind of bias that can improve tactical decisions under pressure [126,155,156,157].

Taken together, these strands support a coherent model: anodal stimulation over task-relevant motor regions eases local inhibitory constraints and strengthens motor-network coupling during practice; cerebellar stimulation accelerates the adaptation component of learning while M1/PMd stimulation fortifies retention and offline performance; and prefrontal stimulation reweights frontostriatal decision circuitry—including dopaminergic tone—to favor efficient, less risky action selection and faster credit assignment. The net result is quicker skill acquisition, better day-to-day carry-over, and more intelligent choices under cognitive or physical load—especially when montage, dose, and timing are tuned to the specific skill being trained and the consolidation window that follows.

## 6. Limitations

The evidence base synthesized in this review is constrained by several methodological and reporting limitations that complicate interpretation and reduce confidence in between-study comparisons. A recurring issue is the generally small sample size of many included trials, with several studies recruiting fewer than 20–30 athletes and, in some cases, relying on even smaller analytic subsamples for specific outcomes (e.g., biochemical markers). These modest samples limit statistical power, inflate the risk of type II error, and make effect estimates unstable—particularly when outcomes are inherently variable in sport settings (e.g., recovery perceptions, match-to-match load, or skill execution). Small samples also restrict the ability to examine moderators such as sex, age group, playing position, or baseline performance level, even though the included studies span adolescents to adults, amateur to elite players, and male and female athletes. Addressing these issues will enable future meta-analyses to be conducted that include tDCS studies in soccer players.

A second limitation is substantial heterogeneity in both participant characteristics and intervention contexts. Although most samples fall within a relatively narrow range of height, body mass, and BMI, the studies differ meaningfully in competitive standard, training history, weekly training exposure, and whether testing occurred under match-related fatigue, post-match recovery, or routine training conditions. These contextual differences are significant in soccer, where acute fatigue and cumulative training load strongly influence physical outputs, perceptual-cognitive performance, and subjective recovery. In several studies, the experimental sessions occurred after official matches or fatigue protocols, whereas others were embedded within multi-week training programs; consequently, observed effects may reflect interactions between tDCS and fatigue, learning, or training adaptation rather than a direct stimulation effect alone. In addition, generalizability is limited because many investigations were recruited from a single team, a single academy, or a single university setting, often within one country, which increases the risk that local training culture, coaching practices, or recovery routines shape outcomes.

Intervention heterogeneity further limits synthesis. Across studies, tDCS targets varied (DLPFC, M1, premotor regions), electrode montages differed (bilateral cephalic placements vs. extracephalic references), session dose ranged (10–30 min; ~1.5–2 mA), and protocols spanned single-session designs to repeated stimulation over weeks. Such variation changes the likely distribution of current in the brain and may lead to qualitatively different mechanisms (e.g., executive control modulation versus corticospinal excitability), making it difficult to treat “tDCS” as a uniform exposure. This concern is amplified by limited reporting on dose-related factors such as electrode preparation, impedance, and, most importantly, individualized estimates of cortical electric field. Without head-anatomy-informed modeling (or at least proxies like scalp-to-cortex distance), it is not possible to determine whether null findings reflect true inefficacy or simply insufficient/variable brain dosing across participants.

Outcome selection and measurement add another layer of limitation. The included studies used a wide range of endpoints–from well-being and perceived recovery scales to heart-rate indices, jump tests, isometric strength, balance metrics, and perceptual-cognitive tasks. This breadth is valuable, but it fragments the evidence and makes cross-study aggregation difficult, especially when outcomes differ in sensitivity and susceptibility to learning effects. Some commonly used physical tests (e.g., CMJ) may show ceiling effects in well-trained athletes or may be insensitive to short-term neuromodulatory changes, whereas subjective outcomes can improve due to expectancy, placebo effects, or regression to the particularly in crossover designs conducted in competitive seasons. Several studies reported improvements over time in both active and sham conditions, highlighting the challenge of disentangling stimulation effects from natural recovery, familiarization with tests, or concurrent recovery interventions.

Risk of bias is also a significant limitation. While many studies implemented sham stimulation and described blinding attempts, blinding integrity was not continually assessed, or may be compromised in practice because sensations can differ between active and sham conditions. Some investigations were single-blinded or quasi-experimental rather than fully randomized controlled trials, and at least one study relied on retrospective season-to-season comparisons, which increases vulnerability to confounding from unmeasured changes in training, scheduling congestion, squad rotation, or medical practices. In multi-component interventions combining tDCS with stability training, HIIT, sling exercises, visuomotor training, or pneumatic compression, attribution is inherently uncertain: improvements may arise from the training stimulus itself, from the interaction of stimulation with training, or from differences in adherence, coaching emphasis, or progression across groups.

Finally, reporting limitations reduce interpretability. Anthropometrics and body composition were inconsistently reported, and key descriptors—such as playing position distribution, menstrual cycle/contraceptive status in female cohorts, sleep, nutrition, caffeine use, or detailed match load—were frequently absent despite their relevance to recovery and performance. Adverse events were typically described as mild, but standardized reporting frameworks were seldom used, and longer-term follow-up was rare, leaving uncertainty about durability and safety across congested competitive periods. Taken together, these limitations suggest that the current literature is best interpreted as suggestive and context-dependent, with promising signals in some recovery, skill, and perceptual-cognitive domains, but insufficient methodological consistency and dosing characterization to draw firm conclusions about effectiveness across soccer populations and performance outcomes.

## 7. Conclusions

Across 21 trials, the soccer-specific evidence suggests—rather than definitively demonstrates—that tDCS effects are small, context-dependent, and sensitive to target × task × timing. Evidence is most consistent for prefrontal (left-DLPFC-dominant) stimulation supporting post-match recovery status/well-being and specific decision-efficiency outcomes under fatigue. However, subjective scales may be placebo-sensitive and require replication with standardized procedures. In contrast, single-session motor-cortex protocols show mixed-to-null effects on rapid force–power performance, while training-embedded, multi-session applications more often report improvements in neuromuscular adaptation (strength, jump/sprint/agility, EMG patterns). Given small samples and heterogeneous endpoints, these findings should be interpreted as a mapped signal and a research agenda, not as generalizable efficacy for routine use.

## Figures and Tables

**Table 1:** Studies included in the review.

Key Findings (vs. Sham/Control)	Outcomes	Timing	Comparator	Dose	Montage/Target	Design/Blinding	Participants	Study
WBQ ↑ more with active tDCS (ES 1.02 vs. 0.53; pre 17.17 → 20.25). TQR ↑ similarly; no between-cond diff.	WBQ (fatigue, sleep, soreness, stress, mood), TQR	14–16 h post-match; next-morning follow-up	Sham (30 s)	2 mA × 20 min; 4 sessions total (2 active, 2 sham)	Anode F3 (L-DLPFC), Cathode F4 (R-DLPFC)	Randomized, double-blind, sham-controlled, counterbalanced crossover; repeated-measures	13 world-class pro women; mean ~26 years old; Brazil; played ≥50% of 4 matches	[55]
No significant differences; trends ↑ for back/leg strength, long jump, sit-ups with active tDCS.	Anthropometrics; flexibility; handgrip; back/leg strength; standing long jump; vertical jump; 1 min sit-ups/push-ups	Resting during stimulation; pre-testing	Sham (30 s)	1.98 mA × 20 min; 2 arms with 2 week washout	Halo Sport; Anode Cz; Cathodes C5, C6 (motor cortex)	Single-blinded, randomized, crossover, sham-controlled	20 elite U-15 boys; Malaysia	[56]
WBQ ↑ in both; no between-cond diff. HRex ↔; HRR and HRR index improved in both; no diff.	WBQ; submax running test 10 km/h: HRex (last 30 s), HRR 60 s, HRR index	Morning ~12–13 h post-match	Sham (30 s)	2 mA × 20 min; 2 conditions	Anode F3 (L-DLPFC), Cathode F4	Single-center, randomized, crossover, double-blind, sham-controlled	12 elite U-20 men; Brazil	[57]
No significant differences for any variable.	Countermovement jump (height, power); HR; RPE (CR-10); VAS pain; SRS recovery	Post-warm-up/pre-CMJ; repeated across 3 experimental sessions	Sham (30 s) and control (rest)	2 mA × 15 min	Bi-hemispheric anodal M1; electrode over Cz; cathodes on inion	Triple-blinded, randomized, 3-arm (active, sham, control)	27 male U20; mean ≈ 18 years old	[58]
Accuracy ↔; Decision-making RT ↓ with a-tDCS (≈655 → 626 ms). Visual search: more fixation/s and shorter durations (more efficient).	Decision-making (SSG + screen task: accuracy and RT); eye-tracking (fixation/s, fixation duration)	During regular training block	Sham (30 s)	2 mA × 15 min; 40 sessions (5/week × 8 weeks)	Anode F3 (L-DLPFC); Cathode right shoulder (extracephalic)	Pair-matched, randomized, parallel (a-tDCS vs. sham); 10 week protocol	23 male pros (Brazil 3rd division); mean 22.6 years old	[59]
CRT ↓ greater in RF (trained LL) and triceps (untrained UL) with active tDCS; no cognitive differences.	EMG-based choice reaction time (CRT) in RF, VM, AD, BB, Triceps; TMT; Digit Span	tDCS concurrent with visuomotor training	Sham (30 s) + same VMT	2 mA × 20 min; 5 sessions (24 h apart) with VMT during 5–15 min	Anode F3 (L-DLPFC); Cathode Fp2	Randomized, placebo-controlled, double-blind, parallel	30 male amateurs; 18–30 years old	[60]
Dominant limb MVIC ↑ 5.2% (during), 6.3% (30′), 9.4% (60′) with active tDCS; non-dominant ↔.	Quadriceps MVIC (dominant and non-dominant)	Strength tested at T0, T20 (during), T50 (30 min post), T80 (60 min post)	Sham (30 s)	2 mA × 20 min	Anode C3/C4 contralateral M1; Cathode ipsilateral supraorbital	Randomized, double-blind, crossover (1 week washout)	20 female soccer; 15–17 years old; ~5.2 years old training	[61]
Greater ↑ in trunk EMG and jump height with tDCS + exercise (all *p* < 0.05).	EMG: erector spinae, rectus abdominis, external oblique; CMJ (hands-on-hips and with arm swing)	During 30 min stability sessions	Exercise only	2 mA × 20 min; 8 weeks, 3×/week	Halo Sport; C3, C4, Cz (M1)	Randomized, parallel (tDCS + lumbar stability vs. exercise only)	30 male university players	[62]
tDCS > control for ↑ muscle activation and balance metrics (ANCOVA *p* < 0.05).	EMG (%MVIC) RF, BF; Balance (sway area, path length, limit of stability)	After plyometrics each session	Action observation training	2 mA × 20 min; 8 weeks, 5×/week	Halo Sport; Anodes Cz/C3/C4; Cathodes C5/C6	Randomized, parallel (tDCS vs. action observation) with shared plyometrics	30 male university players; Mokpo, Korea	[63]
M1 ↑ Stroop incongruent accuracy; DLPFC → riskier choices (lower IGT net). 2-back ↔.	Stroop; Iowa Gambling Task; 2-back	After maximal cycling to exhaustion (fatigue state)	Sham (30 s)	2 mA × 10 min	Dual-site anodal: M1 (C3 + C4) or DLPFC (F3 + F4); cathodes on shoulders	Counterbalanced within-subject; 3 sessions (M1, DLPFC, sham); double-blind	23 elite national-level (8F); mean ~20.6 years old	[64]
Faster RT in VST (3 and 6 stimuli). Higher ACC in PSBT at −240 ms. Other measures ↔.	VST (RT/ACC), PSBT (RT/ACC), PSPT (RT/ACC) across difficulty/time offsets	Post-fatigue (60% Powermax cycling)	Sham	2 mA × 10 min	Left PMd (premotor dorsal) anodal; 2 mA	Repeated-measures; balanced anodal vs. sham after induced sports fatigue	24 high-level (6F); mean 20.1 years old	[65]
Large improvements only in tDCS: RT 354 → 256 ms; pass accuracy 46% → 68%. Sham/control ↔.	Reaction time (visual/auditory simple and choice); match passing accuracy (%)	Immediately before training each day	Sham (30 s) and control	1.5 mA × 15 min; 6 consecutive days	Anode C3 (M1); Cathode Fp2	Quasi-experimental pre/post; randomized to tDCS, sham, control	36 skilled men; Tehran; 3 groups (*n* = 12)	[66]
WBQ ↑ selectively with active tDCS; TQR similar. EMG ↑ (VL peak at 24 h; RF stable) but CMJ ↔. LSPT improved only with active tDCS (−2.4 s MT; errors halved; −7.7 s total).	WBQ; TQR; Stroop interference; CMJ; EMG (VL, RF %MVC); LSPT (movement time, errors, total time, RPE)	After 90 min soccer-match simulation	Sham	2 mA × 20 min per visit (3 total)	Anode F3 (L-DLPFC); Cathode F4	Double-blind, counter-balanced crossover; 3 visits (0 h, +24 h, +48 h) post SMS	16 pro male outfield; mean 20.9 years old	[67]
Added tDCS: PR +12%; sleep +7.5%; soreness −64%; CK −76%; less inter-individual CK variability.	Perceived recovery; sleep; soreness; CK	Recovery days post-match; outcomes on day +2	Pneumatic compression only (prior season)	2 mA × 20 min, day-after-match	Anode F3; Cathode F4 (DLPFC)	Retrospective real-world comparison: 2022 pneumatic compression vs. 2023 pneumatic + tDCS	Pro male first-division team; 2 seasons; *n* = 18 (perceptual), *n* = 9 (CK)	[68]
Earlier/stronger L4–S1 output; emergence of 8th synergy; more temporally focused activations; subtle CI shifts. Interpreted as more efficient control.	sEMG of 14 LL muscles; spinal motor output mapping; muscle synergies (NMF); co-activation index; high-speed video (phase segmentation)	Immediately post-stimulation	Pre vs. post (no sham)	2 mA × 20 min; kicks within 5 min post	Halo Sport; M1 targeting	Within-subject pre/post after single session	20 male national first-class; right-leg dominant; mean 18.5 years old	[69]
All ↑; greater with tDCS: VO_2max_ 57.7 → 61.5 vs. 56.4 → 58.2; shuttle 113.3 → 121.5 vs. 112.8 → 119.4; Yo-Yo 1299 → 1697 m vs. 1279 → 1644 m.	VO_2max_; 20 m shuttle; Yo-Yo IR	During 20 min HIIT sessions	HIIT alone	2 mA × 30 min; 8 weeks, 5×/week	Halo Sport; M1	Randomized parallel (HIIT + tDCS vs. HIIT)	30 male college; 2 groups (*n* = 15)	[70]
Greater gains with tDCS across EMG, sprint, agility (ANCOVA *p* < 0.05).	EMG: erector spinae, rectus abdominis, external oblique; 30 m sprint; *T*-test agility	During sling stabilization sessions	Sling exercise only	2 mA × 30 min; 8 weeks, 2×/week	Halo Sport; C3, C4, Cz (M1)	Randomized parallel (sling + tDCS vs. sling)	30 college men; 2 groups (*n* = 15)	[71]
Flexibility ↑ for skilled and semi-skilled (not amateurs). Social decision-making ↑ across all levels.	WCST (cognitive flexibility); Ultimatum Game (social decision-making)	Laboratory sessions	Sham	2 mA × 20 min; 3 sessions	Anode right DLPFC (Fp2/F3 area); Cathode contralateral supraorbital	Semi-experimental pre/post; randomized to active vs. sham	60 male players (20 skilled, 20 semi-skilled, 20 amateur)	[72]
Experts improved only with L-anodal/R-cathodal (≈52.3% → 59.9%). Novices: no change.	Video-based soccer decision-making accuracy	Pre-/post- single session	Sham; opposite polarity	2 mA × 20 min	F3/F4 (DLPFC): L-anodal/R-cathodal; R-anodal/L-cathodal; Sham	Randomized, single-blind, sham-controlled; 3 groups	66 (33 experts, 33 novices); mixed sex	[73]
Greater improvements with active tDCS: FPPA +5.65% vs. +2.26%; jump +25.3% vs. +10.4%; 8-hop −21.1% vs. −14.3%.	FPPA (single-leg landing); vertical jump; 8-hop test	tDCS before core stability each session	Sham + same training	2 mA × 15 min; 8 weeks, 3×/week	Anodes C3 and C4 (bilateral M1); Cathode Fp1	Two-arm, double-blind RCT (active tDCS + core vs. sham tDCS + core)	42 male players (18–25 years old) with dynamic knee valgus	[74]
No between-group differences; both groups improved over time. Anxiety inversely correlated with inhibitory control.	HAM-A anxiety; Stroop; Trail Making	Daily sessions	Sham (30 s)	2 mA × 20 min daily × 7 (+1 maintenance)	Anode F3 (L-DLPFC); Cathode F4	Randomized, double-blind clinical trial; 7 consecutive days + day-14 maintenance	23 players with acute anxiety (12 active, 11 sham); 18–40 years old	[75]

Abbreviations used: tDCS (transcranial direct current stimulation); a-tDCS (anodal tDCS). Brain targets and montages: DLPFC (dorsolateral prefrontal cortex); M1 (primary motor cortex); PMd (dorsal premotor cortex); L/R (left/right). F3, F4, C3, C4, Cz, Fp1, Fp2 are scalp sites from the international 10–20 EEG system (frontal, central, prefrontal positions). Outcomes and physiology: WBQ (Well-Being Questionnaire); TQR (Total Quality Recovery scale); HRex (heart rate during exercise, typically lasting 30 s); HRR (heart rate recovery, often at 60 s); EMG (electromyography; sEMG = surface EMG); MVIC (% maximum voluntary isometric contraction); %MVC/%MVIC (percent of maximum voluntary [isometric] contraction). Muscle labels: RF (rectus femoris), VM (vastus medialis), VL (vastus lateralis), BF (biceps femoris), AD (anterior deltoid), BB (biceps brachii). Subjective scales: RPE (rating of perceived exertion; CR-10 scale when noted); VAS (visual analogue scale for pain); SRS (Subjective Recovery Scale). Performance/cognition: CMJ (countermovement jump); VMT (visuomotor training); CRT (choice reaction time); TMT (Trail Making Test); WCST (Wisconsin Card Sorting Test); UG (Ultimatum Game); LSPT (Loughborough Soccer Passing Test); SSG (small-sided games); HIIT (high-intensity interval training); FPPA (frontal plane projection angle); Yo-Yo IR (Yo-Yo Intermittent Recovery test). Statistics: ES (effect size; Cohen’s *d* unless partial η^2^ is stated); ANOVA/MANOVA/ANCOVA (analysis of variance/multivariate ANOVA/analysis of covariance); RT (reaction time); ACC (accuracy); *p* (*p*-value). Study/admin: SMS (soccer-match simulation); CK (creatine kinase); AEs (adverse events); U-20 (under-20 age group); ↑: increase (higher value)/improvement; ↓: decrease (lower value)/deterioration; →: change from pre to post (e.g., baseline → follow-up); ↔: no meaningful change/no difference detected.

**Table 2:** RoB-2 risk of bias assessment.

Statistical Power	Sample Size	Blinding Success Rate	Bias in Selection of the Reported Result	Bias in Measurement of the Outcome	Bias Due to Missing Outcome Data	Bias Due to Deviations from Intended Interventions	Bias Arising from the Randomization Process	Study
A priori power: 80% for medium effect η^2^ = 0.07 → *n* = 12 required; study analyzed *n* = 13 (meets target). Likely underpowered for small incremental effects.	*n* = 13 elite female players analyzed (from 24); cross-over (2 active + 2 sham per player) across 4 matches; sessions averaged within condition → effective *N* remains 13.	Participants believed they were stimulated in all sessions (13/13), supporting sham credibility; however, real vs. sham guess accuracy not reported → blinding integrity cannot be quantified.	Some concerns	Some concerns	Low	Low	Low to some concerns	[55]
Power: NR (no sample size calculation; likely underpowered for small effects)	*N*: 20 (cross-over; each participant in both conditions)	Blinding success: NR (no blinding check/guess test)	Some concerns	Some concerns	Some concerns	Some concerns	Some concerns	[56]
Not reported (no a priori power/sample size calculation)	12 male U-20 players analyzed; crossover design; no dropout; each participant received both anodal and sham	Correct condition guesses = 29%; most participants believed they received active stimulation in both sessions → suggests no meaningful unblinding	Some concerns	Low to some concerns (outcome-dependent)	Low risk	Low risk	Low risk	[57]
A priori power analysis (G*Power): MANOVA RM within–between; α = 0.05; assumed effect size = 0.25; target power = 0.96; required *N* = 27.	*N* = 27 total (Active *n* = 9; Sham *n* = 9; Control *n* = 9).	Not reported (no formal blinding check). Active vs. sham intended blinded via independent researcher/device codes; control group unblinded by design	Some concerns	Some concerns	Low	High	Low	[58]
Final analyzed *n* = 23, which remains above the planned *n* = 20; however, power for each specific outcome (e.g., fixations, RT) was not separately reported	*n* = 23 analyzed (a-tDCS *n* = 11; sham *n* = 12); 3 excluded (attendance/injury)	Not reported (no formal blinding check)	Some concerns	Low to some concern	Some concerns	Some concerns	Some concerns	[59]
A priori power target: 80% (β = 0.20) for primary rectus femoris CRT difference. Reported post hoc test’s power across outcomes ranged 0.08–0.98 (rectus femoris 0.98; vastus medialis 0.96; triceps 0.69; cognitive outcomes low: TMT-A 0.12, TMT-B 0.08, DSF 0.25, DSB 0.28	*N* = 30 randomized (15/group); sample size calculated in G*Power for ANCOVA (α = 0.05, β = 0.20) assuming minimal between-group ΔCRT = 0.07 (SD = 0.05) for rectus femoris; achieved *N* = 30 with 0% attrition.	Blinding check performed at final session; all participants reported believing they were stimulated during all sessions. Allocation-guess accuracy (% correct) not reported; blinding success rate not quantifiable.	Some concerns	Low (primary CRT); Some concerns (secondary cognitive tests)	Low	Some concerns	Some concerns	[60]
No a priori power calculation reported. Post-hoc approx. power with *n* = 20 (paired): ~0.72 for *d* = 0.6; >0.96 for d ≥ 0.9 (dominant limb effect sizes reported)	*N* = 20 female adolescent soccer players; randomized crossover with 7-day washout; 20/20 completed, no missing outcome data reported	Double-blind stated (participants and evaluator blinded); sham used 30 s stimulation to mimic sensations; blinding success not assessed/reported (no guess test/blinding index)	Some concerns	Some concerns	Low	Low	Low	[61]
Not reported (no a priori power/sample size calculation provided)	*N* = 30 total; 15 per group (tDCS + lumbar stability exercise *n* = 15; lumbar stability exercise control *n* = 15)	Not reported (no sham tDCS described; blinding assessment not performed/NR)	Some concerns	Some concerns	Some concerns	High	Some concerns	[62]
NR—No a priori power/sample size calculation reported (α = 0.05 stated only).	*N* = 30 (reported as 15/15); baseline table lists control *n* = 14—unclear attrition/reporting discrepancy	NR—No sham condition and no blinding-success assessment reported; participant blinding unlikely	Some concerns	Some concerns	Some concerns	High	Some concerns	[63]
A priori power calculation not reported; therefore, achieved statistical power cannot be verified from the manuscript	*N* = 23 elite soccer athletes; within-subject crossover with 3 sessions (M1, DLPFC, sham); no dropouts reported	Not reported quantitatively. Participants completed a post-session questionnaire on stimulation sensations/perceptions, but the paper does not report the proportion correctly guessing real vs. sham (or any blinding index)	Some concerns	Low	Low	Some concerns	Some concerns	[64]
A priori power calculation not reported. With *N* = 24 in a within-subject comparison, the study is typically powered for medium-to-large effects; small effects likely underpowered	*N* = 24 high-level soccer athletes (age 20.1 ± 1.8 years old; training experience 9.9 ± 2.9 years old; females; non-goalkeepers)	Not reported (no blinding assessment/guess test described)	Some concerns	Low	Some concerns	Some concerns	Some concerns	[65]
Total sample is small (~12/group), so power for small/moderate effects is likely limited; observed effects were large (ANCOVA partial η^2^ reported)	Enrolled *N* = 36; allocated 12/12/12 (M1 tDCS/sham/control)	Participant blinding attempted using sham (current stopped after 30 s without informing participants); outcome assessors (coaches rating performance videos) were blinded (single-blind)	Some concerns	Low to some concerns	Some concerns	High	Some concerns	[66]
A priori powered at 0.80; reported observed power across outcomes ranged approximately 0.06–1.00, with highest power for significant LSPT/EMG effects and lower power for several null findings	Randomized, crossover; *n* = 16 male professional/elite U-21 soccer players. G*Power repeated-measures ANOVA within factors: α = 0.05, power = 0.80, effect size f = 0.4, corr = 0.5 → required *n* = 12; recruited *n* = 16 to account for dropout.	Correct-guess rate differed by condition: active sessions 81.3%, 87.5%, 100% vs. sham sessions 18.8%, 6.3%, 12.5%. Authors note most participants (>80%) guessed they received active stimulation in both conditions (reported as “active stimulation guess rate”), concluding that blinding was effective.	Some concerns	Low to some concerns	Low	Some concerns	Low	[67]
A priori power calculation: Yes (G*Power 3.1.9.4; ANCOVA; two-sided α = 0.05; power = 0.80; effect size = 0.50). Required *N* = 28, inflated to *N* = 30 to allow for attrition.	Total randomized/enrolled: *N* = 30; Per group: 15 (HIIT + tDCS), 15 (HIIT); Attrition/completion: 0 dropouts; 100% completion/attendance reported	Not reported/Not assessed. No sham tDCS described; participants likely aware of allocation (Halo Sport headset in tDCS group only)	Some concerns to high	Some concerns	Low	High	Some concerns	[70]
A priori target power: 0.80 (G*Power; ANCOVA; α = 0.05; effect size f = 0.50). Post-hoc/achieved power not reported.	*N* = 30 total (15 per group). A priori sample size calculated with G*Power (ANCOVA; α = 0.05; power = 0.80; effect size = 0.50) indicated *N* = 28; inflated to *N* = 30 to account for dropout; 0 dropouts (100% completion).	Not assessed/not reported. No sham tDCS procedure described; blinding success (e.g., guess of group assignment) was not measured	Some concerns	Some concerns	Low	High	Some concerns	[71]
A priori power/sample size calculation: Not reported; Observed (post-hoc) power reported in ANCOVA tables: Cognitive flexibility: Power 1.00 for group effect; 0.87 for pre-test covariate. Social decision-making: Power 1.00 for group effect; 1.00 for pre-test covariate.	*N* = 60 male football players (Mage ≈ 24.27);Real tDCS *n* = 30; sham tDCS *n* = 30; Each arm: 10 skilled, 10 semi-skilled, 10 amateur; attrition not reported; analyses suggest *N* = 60 included	Not reported/not assessed (no participant or assessor guess questionnaire reported). Sham described as 30 s stimulation then off.	Some concerns	Low to some concerns	Low	Some concerns	Some concerns	[72]
A priori power analysis reported (G*Power): target power = 0.80, α = 0.05, assumed medium effect size f = 0.25, repeated-measures within–between interaction, 6 groups, 2 measurements, r = 0.5; required *N* = 60; recruited *N* = 66 to allow for dropout.	*N* = 66 total (33 experts, 33 novices). Randomized into 3 groups *n* = 22 each, with 11 experts + 11 novices per group.	Not reported/not assessed. Study states participants were kept blind to stimulation conditions, but no formal blinding check (e.g., condition-guess questionnaire or blinding index) and no % correctly guessing condition were provided.	Some concerns	Low	Low	Some concerns	Some concerns	[73]
A priori power calculation using G*Power: 80% power (1 − β = 0.80), α = 0.05 (two-tailed), assumed effect size *d* = 0.80 → minimum 21/group. Achieved sample met target; post hoc power not reported	Sample size (planned/actual): A priori target *N* = 42 (21/group); actual enrolled and analyzed N = 42 (21 active, 21 sham); 0 dropouts	Blinding assessed via post-intervention questionnaire (participants guessed active vs. sham), but numerical results (e.g., % correct guesses/Bang blinding index) were not reported → blinding success unclear	Some concerns	Low	Low	Low	Low	[74]
Power/sample size calculation: Not reported. Statistical power unclear; final analyzed sample was small (*n* = 23), with substantial attrition.	Randomized: *n* = 47 (anodal tDCS *n* = 24; sham *n* = 23). Analyzed (final): *n* = 23 (anodal *n* = 12; sham *n* = 11). Attrition after randomization: 24/47 (~51%)	Blinding success rate: Not assessed/not reported. Study describes double-blinding procedures (device behind participant; no sound; assessors blinded), but no post-trial blinding check (e.g., guess allocation) was reported	Some concerns	Low	High	Low	Some concerns	[75]

→: leads to/therefore/implies.

**Table 3:** ROBINS-I risk of bias assessment.

Statistical Power	Sample Size	Blinding Success Rate	Bias in Selection of the Reported Result	Bias in Measurement of Outcomes	Bias Due to Missing Data	Bias Due to Deviations from Intended Interventions	Bias in Classification of Interventions	Bias in Selection of Participants into the Study	Bias Due to Confounding	Study
Not reported. No a priori sample size calculation or statistical power analysis described; no post-hoc power provided.	*N* = 18 players included in perceptual outcomes (perceived recovery, sleep quality, muscle pain/soreness); *N* = 9 players for CK analysis. Data derived from 10 matches (2022) and 10 matches (2023).	Not applicable/not assessed. No sham condition; participants and staff were not blinded. Blinding success was not measured	Moderate	Serious	Moderate to serious risk	Serious	Low	Serious	Critical	[68]
Not reported. No a priori sample-size or power calculation described; power is unknown (study appears exploratory/pilot).	*N* = 20 male national first-class soccer players. Each participant performed 3 penalty-kick trials pre-intervention and 3 post-intervention.	Not reported/Not assessed. No sham/control; blinding procedures not described. Blinding success rate cannot be calculated	Serious	Moderate	Low/Unclear	Serious	Low	Moderate	Critical	[69]

**Table 4:** Evidence tiers by athlete level (soccer-specific tDCS trials).

Professional/Elite	Collegiate/Sub-Elite	Youth/Adolescent	Outcome Domain (Typical Target/Context)
Limited: WBQ improvements beyond sham reported in some controlled recovery-window studies, but not consistently across all elite designs	Insufficient	Insufficient–Limited (few youth studies in true post-match contexts)	Post-match recovery well-being (WBQ domains; DLPFC +F3/−F4; recovery-window)
Insufficient: typically improves over time similarly in active and sham conditions	Insufficient	Insufficient	Global recovery ratings (TQR/SRS; DLPFC or M1; recovery-window/acute)
Limited: supportive real-world season comparison suggests CK reduction, but non-randomized/confounded	Insufficient	Insufficient	Biochemical recovery (CK; DLPFC +F3/−F4; applied recovery program)
Insufficient: well-controlled acute studies generally show no CMJ benefit	Insufficient	Insufficient	Immediate explosive power (CMJ; mostly M1 montages; single-session)
Insufficient	Insufficient	Limited: limb-specific MVIC increases shown in one controlled adolescent sample	Immediate isometric strength (MVIC; unilateral M1; single-session)
Insufficient–Limited: very few multi-week RCTs in true elite/pro squads; generalizability uncertain	Confirmed: multiple RCT-style multi-week programs show larger gains than training alone (jump, sprint/agility, aerobic indices, EMG)	Insufficient	Training-embedded neuromuscular adaptation (jump/sprint/agility/endurance; M1-centric; multi-week paired with training)
Limited: expert-only accuracy improvement under specific polarity; technical passing test improvements reported in controlled recovery protocol; evidence not yet replicated broadly	Limited: some studies show improvements, but designs/outcomes vary	Insufficient	Decision speed/perceptual efficiency (RT/CRT; usually DLPFC with repetition and/or practice; sometimes M1 under fatigue)
Limited: expert-only accuracy improvement under specific polarity; technical passing test improvements reported in controlled recovery protocol; evidence not yet replicated broadly	Limited: some studies show improvements, but designs/outcomes vary	Insufficient	Decision accuracy/soccer-specific technical execution (video decision accuracy; passing tests e.g., LSPT; montage-dependent)
Insufficient: often unchanged despite other benefits; fatigue state may alter direction (e.g., M1 helps Stroop under fatigue)	Insufficient	Insufficient	Executive function tests (Stroop/TMT/Digit Span; mixed targets/contexts)
Confirmed	Confirmed	Confirmed (mild transient sensations; no serious AEs in included trials)	Safety/tolerability

## Data Availability

No new data were created or analyzed in this study. Data sharing is not applicable to this article.

## Supplementary Materials

The following supporting information can be downloaded at: https://www.mdpi.com/article/10.3390/jcm15031281/s1, Table S1: PRISMA-ScR Checklist, reference [158] is cited in the Supplementary Materials.
